# Characterization of gut symbionts from wild-caught Drosophila and other Diptera: description of Utexia brackfieldae gen. nov., sp. nov., Orbus sturtevantii sp. nov., Orbus wheelerorum sp. nov, and Orbus mooreae sp. nov.

**DOI:** 10.1099/ijsem.0.006516

**Published:** 2024-09-27

**Authors:** Laila E. Phillips, Kathleen L. Sotelo, Nancy A. Moran

**Affiliations:** 1 Department of Integrative Biology, The University of Texas at Austin, Austin, TX 78712, USA

**Keywords:** *Drosophila*, host specificity, host-associated microbiota, insect microbiome, *Orbaceae*, symbiosis

## Abstract

Non-culture based surveys show that the bacterial family *Orbaceae* is widespread in guts of insects, including wild *Drosophila*. Relatively few isolates have been described, and none has been described from *Drosophila*. We present the isolation and characterization of five strains of *Orbaceae* from wild-caught flies of the genera *Drosophila* (Diptera: Drosophilidae) and *Neogriphoneura* (Diptera: Lauxaniidae). Cells are generally rod-shaped, mesophilic, and measure 0.8–2.0 µm long by 0.3–0.5 µm wide. Optimal growth was observed under ambient atmosphere. Reconstruction of phylogenies from the 16S rRNA gene and from single-copy orthologs verify placement of these strains within *Orbaceae*. Cells exhibited similar fatty acid profiles to those of other *Orbaceae*. Strain lpD01^T^ shared 74% average nucleotide identity (ANI) with its closest relatives *Ca.* Schmidhempelia bombi Bimp and *Zophobihabitans entericus* IPMB12^T^. Results from multiple genome-wide similarity comparisons indicate lpD01^T^ should be classified as a novel species within a novel genus. The major respiratory quinone for lpD01^T^ is ubiquinone Q-8. lpD02^T^, lpD03, lpD04^T^, and BiB^T^ are more closely related to *Orbus hercynius* CN3^T^ (76, 77, 76, and 77% ANI, respectively) than to other described *Orbaceae*. Genomic and phylogenetic analyses suggest that lpD03 and lpD04^T^ belong to the same species and that lpD02^T^, lpD03/lpD04^T^, and BiB^T^ are each novel species of the genus *Orbus*. The proposed names of these strains are *Utexia brackfieldae* gen. nov., sp. nov. (type strain lpD01^T^ =NCIMB 15517^T^ =ATCC TSD-399^T^), *Orbus sturtevantii* sp. nov (type strain lpD02^T^ =NCIMB 15518^T^ =ATCC TSD-400^T^), *Orbus wheelerorum* sp. nov. (type strain lpD04^T^ =NCIMB 15520^T^ =ATCC TSD-401^T^), and *Orbus mooreae* sp. nov (type strain BiB^T^=NCIMB 15516^T^ =ATCC TSD-402^T^). The isolation and characterization of these strains expands the repertoire of culturable bacteria naturally associated with insects, including the model organism *D. melanogaster*.

## Introduction

The fruit fly *Drosophila melanogaster* is a favoured model for animal genetics, and more recently a model for gut microbiome research due to its compositionally simple and experimentally tractable gut microbiota [1]. However, little is known about the natural gut microbiome of Diptera and the molecular bases of colonization of the dipteran gut by commensal bacteria. Most experimental studies concerning the *Drosophila* microbiome have been performed using bacteria that are widespread in different environments and that are associated with lab-reared flies but less common in natural populations. As a result, the isolation of host-restricted bacteria that are naturally associated with flies is a welcome supplement to the *D. melanogaster* model system, and even more beneficial when they are amenable to genetic engineering and other experimental manipulations.

Non-culture-based studies of gut microbiomes of wild Diptera, particularly of the genus *Drosophila*, indicate a high relative abundance of bacteria in the family *Orbaceae* [2,5]. The first member of this family was described by Volkmann *et al.* [6] upon isolation of *Orbus hercynius* CN3^T^ from wild boar faeces [6]. The family *Orbaceae* with *O. hercynius* as the type species was later proposed by Kwong and Moran [Bibr R7]following isolation of *Gilliamella apicola* from the honey bee, *Apis mellifera* [7]. Subsequent non-culture-based sampling of gut microbiotas from a variety of insect taxa has revealed a specific association between *Orbaceae* and insects, and studies of *Gilliamella* spp. and honeybees demonstrate the experimental potential of this family [8,12].

Over a dozen species of *Orbaceae* have been formally characterized and described [6,7, 13,19], but none has been isolated from a fly. Studying *Orbaceae* in *D. melanogaster* may facilitate the identification of molecular mechanisms governing colonization, host specificity, and interbacterial dynamics of commensal bacteria, due to the availability of genetic tools for hosts and their potential development for these symbionts.

Here we describe five strains of bacteria in the family *Orbaceae* newly isolated from wild-caught flies.

## Methods

### Isolation

Flies were collected using baits of fermented banana and yeast [20] between 2018 and 2022 at Brackenridge Field Laboratory and a residential area in Austin, Texas, USA. Flies were photographed and surface sterilized with 10% w/v sodium hypochlorite. Wings and legs were removed and stored in 95% v/v ethanol for subsequent DNA extraction to confirm species identity of flies. Single flies were homogenized in Insectagro (Corning, AZ, USA) using a sterile micropestle and serial dilutions of the homogenate were plated onto heart infusion agar (HIA) with 5% v/v defibrinated sheep’s blood (HIAb) and incubated at 30 °C and 5% CO_2_. Single colonies with a morphology resembling other *Orbaceae* (transparent perimeter with an off-white centre in a ‘bullseye’ pattern) were inoculated in Insectagro and subsequently stored as glycerol stocks (20% v/v) at −80 °C.

### Host identification by mitochondrial DNA barcoding

DNA was extracted from preserved wings and legs using the supplementary protocol ‘Purification of total DNA from insects using the DNeasy Blood and Tissue Kit’ from the DNeasy Blood and Tissue Kit (Qiagen, Venlo, Netherlands). PCR amplification of mitochondrial genes was performed using primers LCO1490 (5′-GGTCAACAAATCATAAAGATATTGG-3′; forward) and HCO2198 (5′-TAAACTTCAGGGTGACCAAAAAATCA-3′; reverse) [21] for cytochrome oxidase I (COI), and primers A-tLEU (5′-ATGGCAGATTAGTGCAATGG-3′; forward) and B-tLYS (5′-GTTTAAGAGACCAGTACTTG-3′; reverse) [22] for cytochrome oxidase II (COII). COI PCR amplicons were Sanger sequenced at the Genomic Sequencing and Analysis Facility (GSAF) at the University of Texas at Austin (UT-Austin, USA). COII PCR amplicons were nanopore-sequenced (Oxford Nanopore Technologies [ONT], Oxford, UK) by Plasmidsaurus (OR, USA). COI and COII sequences were aligned to the NCBI nt database using the Basic Local Alignment Search Tool (blast) [23], and COI sequences were also aligned to the Barcode of Life Data System (BOLD) database [24].

### 16S rRNA gene phylogeny

Initial phylogenetic analysis was performed using 16S rRNA gene amplicons. Bacterial lysates were used as the template for PCR amplification of an *Orbaceae*-specific ~750 bp region of the 16S rRNA gene using primers 27f-CM (5′-AGAGTTTGATCMTGGCTCAG-3′; forward) [25] and *Orbaceae*-specific primer Orb742R (5′-ATCTCAGCGTCAGTATCTGTCCAGAA-3′; reverse) [26]. Amplicons were purified using the QiaQuick PCR Purification Kit (Qiagen) and submitted for Sanger sequencing at the UT-Austin GSAF as described previously. Sequences were trimmed in Geneious Prime (v. 2021.1) and aligned to the NCBI nt database using blast [23]. Isolates yielding sequences closest to *Orbaceae* 16S rRNA sequences were inoculated in Insectagro or BD BBL brain heart infusion broth (BHI; Becton, Dickinson and Company, MD, USA) and grown for 2 days at 30 °C and 5% CO_2_. Genomic DNA was extracted from these cultures using the ‘Pretreatment for Gram-Negative Bacteria’ and ‘Purification of Total DNA from Animal Blood or Cells’ protocols for the DNeasy Blood and Tissue Kit (Qiagen). A longer portion of the 16S rRNA gene (~1450 bp) was amplified via PCR with universal bacterial 16S rRNA primers 16SA1 (5′-AGAGTTTGATCMTGGCTCAG-3′; forward) and 16SB1 (5′-TACGGYTACCTTGTTACGACTT-3′; reverse) [27] and amplicons were purified as described previously. Amplicons were Sanger-sequenced using the same primers by the UT-Austin GSAF. Trimming and alignment to obtain the consensus sequence for the near-full 16S rRNA gene was performed in Geneious Prime (v. 2021.1). Final, full-length (~1535 bp) 16S rRNA gene sequences were obtained from subsequently assembled genomes using Barrnap (v. 0.9) [28], which agreed with those obtained via PCR amplicon sequencing.

For phylogenetic analysis, *Orbaceae* and outgroup 16S rRNA gene sequences were obtained from NCBI or extracted from RefSeq assemblies using Barrnap (v. 0.9) when 16S rRNA sequences were not available. SSU-Align [29] (v. 0.1.1) was used to align and mask sequences for a final maximum length of 1468 residues. mega [30] (v. 11.0.11) was used to identify the best-fit model, and mega-CC [31] was used to reconstruct a maximum-likelihood phylogeny using the Kimura two-parameter (K2) model with proportion of invariable sites (+I) and gamma distribution (+G) and 1000 bootstrap replicates.

### Genome features

Genomic analyses were performed using whole genome sequence assemblies obtained from long- and short-read sequencing. Glycerol stocks were streaked onto HIAb and grown for 3–4 days. Plates were sent to SeqCenter (Pittsburgh, PA) for DNA extraction, sequencing, and assembly. DNA extraction was performed using the Qiagen DNeasy Blood and Tissue Kit. Genome sequencing was based on a combination of Illumina (San Diego, CA, USA) short reads and nanopore long reads. Libraries were prepared with the ONT Ligation Sequencing Kit (ONT, SQK-LSK109), NEBNext Companion Module (E7180L; New England Biolabs, MA, USA) and ONT Native Barcode Kits (ONT, EXP-NBD104, EXP-NBD114). Samples were run on Nanopore R9.4.1 flow cells and a MinION Mk1B device. Guppy (v. 5.0.16) was used for high-accuracy base calling and demultiplexing [32]. Illumina sequencing was performed as paired-end (2×151 bp) reads on a NovaSeq X. Quality control and adapter trimming was performed using default parameters in the proprietary Illumina software bcl-convert (v. 3.9.3) for Illumina reads and porechop (v. 0.2.3_seqan2.1.1) [33] for ONT reads. Hybrid assembly of Illumina and nanopore reads was performed using Unicycler (v. 0.4.8) [34]. Assembly statistics were recorded using QUAST (v. 5.0.2) [35].

Baseline assembly metrics for the presently-described strains and previously-described *Orbaceae* were calculated using genomes annotated using the RAST Server [36,38]. Annotated assemblies were assessed for quality and completeness using CheckM (v. 1.1.6) [39] and BUSCO (v. 5.4.7) [40,41]. tRNAs were predicted using trnaSCAN-SE (v. 2.0.12) [42] using default options for bacterial genomes. All tRNAs flagged as possible pseudogenes were removed. Barrnap (v. 0.9) [28] was used to extract full ribosomal RNA gene sequences from assembled genomes.

Pairwise average nucleotide identity (ANI) based on BLASTN+ alignments (ANIb) [23,43] and tetranucleotide frequency analysis (TI) were calculated in anvi’o (v. 8.0) [44] using PyANI 0.2.12 [45]. Reported ANIb values are averages of the reciprocal comparisons. *In silico* ddDH values were obtained using the Genome-to-Genome Distance Calculator (GGDC) v. 3.0 [46,47] with the BLAST+ alignment and Formula two options selected via the online portal (https://ggdc.dsmz.de). Pairwise average amino acid identity (AAI) estimation of RAST-annotated assemblies was performed using the online tool AAI-matrix (http://enve-omics.ce.gatech.edu/g-matrix) [48].

Phylogenetic analysis was performed using 856 single-copy orthologs shared between the presently-described strains and other *Orbaceae* for which whole genome sequences were available. *Escherichia coli* ATCC 11775^T^ was included as an outgroup. Assemblies were annotated using the RAST server [36] and orthogroups were identified using OrthoFinder [49] (v. 2.5.5). Single-copy orthologs were aligned using muscle [50] (v. 5.1), and alignments were trimmed using ClipKIT [51] (v. 1.3.0). IQ-TREE (v. 2.2.5) [52] was used to identify the best-fit model via ModelFinder [53] and reconstruct the phylogeny from trimmed alignments. The general amino-acid exchange rate matrix (LG) [54] with empirical amino acid frequencies (+F), proportion of invariable sites (+I), and the FreeRate model of heterogeneity across sites with five categories [55,56] was used. Non-parametric bootstrapping was performed with 1000 replicates.

Genes encoding secretion systems were predicted from RAST-annotated assemblies using MacSyFinder (v. 2.0) via the TXSScan model (v. 1.1.0) [57,60]. Further analyses were performed using anvi’o [44]. Genes and pathways underlying metabolism were predicted using the programme anvi-estimate-metabolism according to Kyoto Encyclopaedia of Genes and Genomes (KEGG) Orthologs (KOs) in the KEGG MODULE database [61,63]. Modules for which ≥75% required genes were detected were considered complete. Pangenome analysis was performed using the programme anvi-pan-genome and annotated according to KEGG, NCBI Clusters of Orthologous Groups of proteins (COGs) [64,65], and Pfam [66,67] databases. Assemblies were annotated with COGs using DIAMOND [68] in sensitive mode. A functional enrichment analysis according to host order was performed on all *Orbaceae* assemblies using the programme anvi-compute-functional-enrichment-in-pan.

### Physiology and biochemical characteristics

Morphology and size were assessed using scanning electron microscopy (SEM). Strains were grown on HIAb for 4 days. Fixative comprised of 2.5% v/v glutaraldehyde in 0.1M sodium cacodylate was added either directly to the colonies on the plates, or a small disc of ACLAR was carefully touched to the colonies to adhere them to the surface. Cells were fixed overnight at room temperature. The following day, colonies remaining on plates were carefully transferred to 1.5 ml tubes, and 1% w/v osmium tetroxide in 0.1M sodium cacodylate was added to all samples. Cells were washed with DI water before adding freshly-prepared and 0.22-µm-filtered 1% w/v thiocarbohydrazide in DI water. Samples were washed before adding the 1% osmium tetroxide solution an additional time, followed by a final wash. Samples were left overnight in DI water at room temperature. The following day, dehydration was performed by sequentially adding ethanol at the following concentrations: 15, 30, 50, 70, 90, 95, and 100% (v/v). Samples were then immersed in 1 : 1 100% ethanol:hexamethyldisilazane (HMDS), then 100% HMDS, and allowed to dry completely. Samples were sputter-coated in 5 nm platinum and palladium. Samples were imaged at 5 kV with a working distance of 8–12 mm on a Zeiss Supra 40VP Scanning Electron Microscope. The purity of each culture used for SEM was confirmed using a plate streak method on HIAb, and expected identities of the cultures were confirmed by 16S rRNA gene amplicon sequencing.

Strains were subjected to various growth experiments to determine the optimal growth conditions and media. Rate of growth and cellular density were taken into consideration when determining optimal ranges. Growth in different liquid media was tested by inoculating wells of a 96-well plate containing 200 µl BHI, LB, trypticase soy broth (TSB), Columbia broth (CB), or Insectagro with 1 µl of cultures adjusted to absorbance OD_600_=1.0. Absorbance was measured following a shaking step every hour for 48 h. In a similar experiment, growth was assessed after 48 h in CB, fastidious anaerobe broth (FAB), liver infusion broth (LIB), M9 minimal media, marine broth (MB), tryptose broth (TB), and TSB. Growth on agar media was tested by streaking a single colony onto the agar surface followed by incubation for 5–7 days at 30 °C. The rate of growth and the size of isolated colonies were taken into consideration when assessing results. Growth under different oxygen conditions was assessed by growing strains on HIAb plates and in 96-well plates containing BHI at 30 and 35 °C in atmospheric O_2_, 5% CO_2_, or inside sealed pouches containing CO_2_ or anaerobic sachets (GasPak EZ Pouch Systems; Becton, Dickinson and Company, MD, USA). A separate experiment testing growth under different agitation conditions (still or 100 r.p.m.) in 5% CO_2_ and atmospheric O_2_ was conducted by inoculating 5 ml cultures of BHI with 10 µl of OD_600_=1.0 overnight culture for each strain and measuring OD_600_ after 24 and 48 h for each condition. NaCl tolerance was determined by measuring the OD_600_ of each strain grown in media supplemented with different concentrations of NaCl. In an initial experiment, NaCl was added to BHI powder already containing 0.5% w/v NaCl, for final concentrations in increments of 1.0% NaCl ranging from 0.5–6.5% NaCl. Strains were grown for 96 h at 30 °C and 5% CO_2_. In a subsequent experiment, strains were grown in 2X YTD (2X yeast extract tryptone medium supplemented with 0.3% w/v dextrose) containing 0.0–3.5% added NaCl in 0.5% increments for 72 h at 30 °C under ambient atmosphere. Optimal pH was determined by measuring absorbance at OD_600_ of each strain grown in BHI adjusted to pH 4.0–10.0 and CB adjusted to pH 6.0–9.0 in 0.5 pH increments every 24 h up to 96 h at 30 °C. Growth at different temperatures was assessed by inoculating BHI with 1 µl of OD_600_=1.0 overnight cultures for each strain and measuring OD_600_ on a Spark 20M plate reader (Tecan, Männedorf, Switzerland) every 24 h up to 72 h.

Strains were assessed for lyophilization tolerance to identify additional culture preservation methods. BHI (5 ml) was inoculated with a single colony of bacteria using a sterile toothpick and grown for 24–48 h. The starting absorbance of these cultures was OD_600_≥0.3. Cultures were separated into 2 ml aliquots and spun at 4 000 r.p.m. for 10 min and resuspended in 500 µl of ATCC Medium 9520. Samples were flash-frozen in liquid nitrogen prior to lyophilization. Cultures were revived after 3 days by adding 0.5 ml BHI and incubating at room temperature for 10 min. Viability was assessed by plating serial dilutions of the culture on HIAb.

Motility was tested by stabbing a single colony into Motility Agar with TTC (Hardy Diagnostics, CA, USA). Tubes were removed from incubation after 5 days and left on a bench at room temperature. Results were confirmed a week later. Follow-up experiments were performed using semisolid Columbia agar (0.25 and 0.30% w/v agar). Briefly, 1.5 µl of 24–48 h cultures were spotted on the centre of 0.25% Columbia agar plates and incubated at 30 °C. A subsequent experiment intended to assess twitching motility using 0.30% Columbia agar was performed; plates were inoculated in the centre by taking a loop of bacterial culture, mixing it into 0.30% Columbia agar, then using a sharp toothpick to stab a blob of the mixture through to the bottom of the plate. The formation of an interstitial colony potentially indicates twitching motility [69].

Biofilm formation in Insectagro was assessed for strains lpD01^T^, lpD02^T^, lpD03, and BiB^T^ compared to biofilm-forming *Snodgrassella alvi* wkB2^T^ by inoculating 180 µl with 20 µl of OD_600_=1.0 culture in a tissue culture-treated 96-well plate (Corning, NY, USA). After incubating for 48 h at 30 °C and 5% CO_2_, media was removed and the plate was washed twice with DI H_2_O before staining each well with 0.1% w/v crystal violet solution (Ward’s Science, NY, USA) for 15 min. The stain was removed, washed four times with deionized H_2_O, and dried for 3 h in a biosafety cabinet. Any residual cells stained with crystal violet were solubilized using 30% w/v acetic acid, and the absorbance was measured at OD_550_.

Oxidase activity was assessed by smearing colonies onto OxiStrips (Hardy Diagnostics, CA, USA) and observing colour changes after incubation at room temperature for 120 s. Strains were considered oxidase-positive if a colour change occurred in less than 30 s, delayed oxidase-positive if a colour change occurred between 30–90 s, and oxidase negative if no colour change was observed. Catalase activity was tested by submerging pelleted cells in 3.0% v/v hydrogen peroxide and observing bubble formation. Activity of nitrate reductase, ornithine decarboxylase, β-galactosidase, urease, and tryptophan deaminase, as well as indole and acetoin production, and glucose, mannose, and xylose fermentation were assessed using the Microgen GN-ID A System (Microgen Bioproducts Ltd, Surrey, UK). Each strain was streaked onto HIAb and grown for 2–4 days until colonies were large enough for isolation. A single colony was suspended in 3 ml 0.85% w/v NaCl and 100 µl of the suspension was added to each well of the microwell test strip. Tests were repeated three times; twice with 48 h incubation periods as suggested by the manufacturer, and once with a 72 h incubation period to determine if possible false negatives occurred due to insufficient incubation time. Strips were incubated for 48 h at 30 °C. Following incubation, the following reagents were added to the appropriate wells: Kovacs’ reagent (Remel, KS, USA), Voges-Proskauer reagents 40% w/v KOH and 5.0% w/v α-naphthol (Remel), and TDA reagent (Oxoid, Hampshire, UK). A nitrate reduction test was performed by adding nitrate reagents A and B (Remel). A small amount of zinc powder (Fisher Scientific, MA, USA) was added to wells of the nitrate reduction test that remained colourless. Glucose, lactose and/or sucrose fermentation, H_2_S, and gas production were assessed using an 8 ml triple sugar iron (TSI) agar slant (Hardy Diagnostics) by stabbing a single colony into the middle of the slant, streaking the agar surface, and incubating for a total of 7 days. Results were checked 1, 5, and 7 days after inoculation. DNase activity was assessed using BD DNAse Test Agar. Briefly, single colonies were streaked in triplicate onto DNAse agar and incubated for 4 days at 30 °C. HCl (1 N) was added to each plate and allowed to sit for ~2 min to observe if clearance zones indicating depolymerized DNA appeared.

Antimicrobial susceptibility was assessed via a minimum inhibitory concentration (MIC) assay in a 96-well plate format as described in Elston *et al.* [26]. Briefly, 2X serial dilutions of carbenicillin, chloramphenicol, kanamycin, spectinomycin, and tetracycline were prepared to test concentrations of 800–3.125 µg ml^−1^ in 100 µl media. Each well was inoculated with 1 µl of OD_600_=1.0 culture.

The presently-described strains except for lpD04^T^ were previously genetically engineered via conjugation [26]*.* We intoduced Pathfinder plasmids into lpD04^T^ via *Escherichia coli* MFD*pir* containing pSL1 or pSL1-GFP as detailed in Elston *et al.* [26].

### Chemotaxonomy

The fatty acid profiles for the presently-described strains were assessed by EMSL Analytical, Inc. (NJ, USA). Briefly, pure colonies were removed from culture plates, and fatty acids were extracted using the Instant FAME method developed for the Sherlock Microbial Identification System by Biolog, Inc. (DE, USA; previously MIDI, Inc.) [70]. Following extraction, fatty acid methyl esters were assessed using an Agilent 6890 series gas chromatography system (Agilent Technologies, Inc., CA, USA) [70].

Respiratory quinone analysis was carried out by DSMZ Services, Leibniz-Institut DSMZ – Deutsche Sammlung von Mikroorganismen und Zellkulturen GmbH, Braunschweig, Germany.

## Results

### Isolation

Five strains were identified as potentially novel species of *Orbaceae* by 16S rRNA gene amplicon and whole genome sequencing. Strains confirmed as *Orbaceae* were grown for 48 h in Insectagro or BHI and amended with glycerol (20% v/v) to create stocks for cryopreservation at −80 °C.

### Host identification by mitochondrial DNA barcoding

Mitochondrial gene sequences from flies from which strains lpD01^T^, lpD02^T^, lpD03, and lpD04^T^ were isolated aligned most closely to sequences associated with *Neogriphoneura sordida*, *Drosophila cardini*, *Drosophila meridiana*, and *Drosophila melanogaster*, respectively. There was insufficient DNA available for molecular identification of the fly from which strain BiB^T^ was isolated, but it was identified as a species in the genus *Drosophila* based on morphology.

### 16S rRNA gene phylogeny

**Fig. 1.**
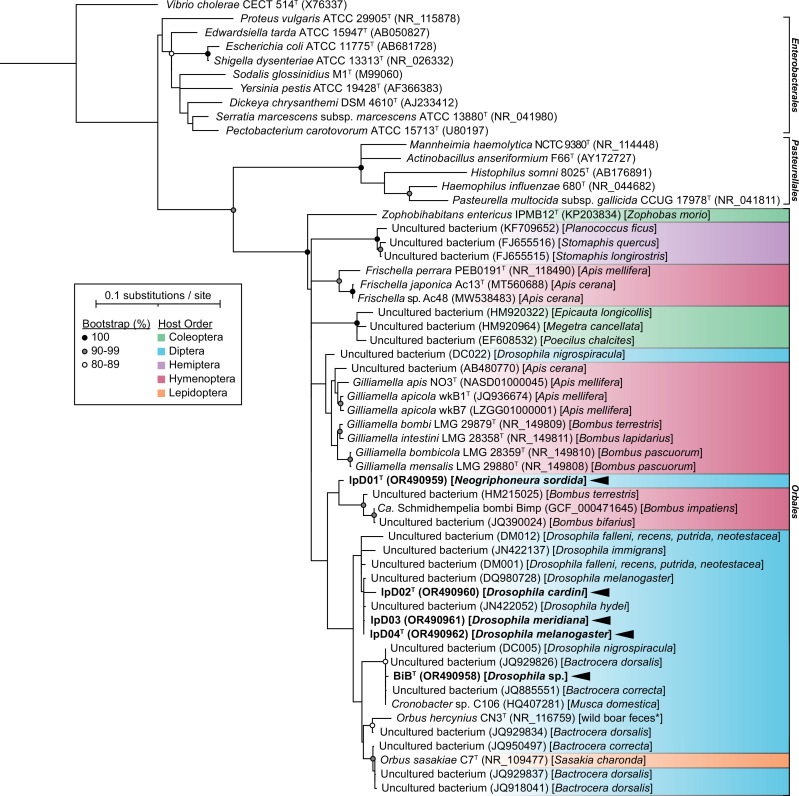
Maximum likelihood phylogeny of the *Orbales* based on the 16S rRNA gene; the *Enterobacterales* and *Pasteurellales* are included as outgroups. The presently-described strains are indicated in bold with an arrow. Parentheses indicate NCBI accession numbers except for DC005 and DC022, which are from reference [3], and DM001 and DM012, which are from reference [2]. The source of isolation is indicated in brackets, and the corresponding host order is indicated according to colour. * = putatively fly-associated [6]. The scale bar represents 0.1 nucleotide substitutions per site.

The 16S rRNA gene phylogeny places strains lpD01^T^, lpD02^T^, lpD03, lpD04^T^, and BiB^T^ within the family *Orbaceae* (Fig. 1). Strain lpD01^T^ forms a distinct lineage within the *Orbaceae*, and its closest relatives are *Ca.* Schmidhempelia bombi Bimp [71] and two other uncultured bacteria from *Bombus* spp. Strains lpD02^T^, lpD03, and lpD04^T^ are closely related to each other, and are part of a lineage of uncultured bacteria associated with *Drosophila* spp. BiB^T^ is part of a lineage of uncultured bacteria associated with *Drosophila* and two other fly genera, *Bactrocera* and *Musca*. Its closest cultured relatives are *Orbus hercynius* CN3^T^ and *Orbus sasakiae* C7^T^. Notably, this phylogeny suggests a general association between distinct lineages of *Orbaceae* and order of the insect host, with few exceptions.

### Genome features

The assemblies for lpD01^T^, lpD02^T^, lpD03, lpD04^T^, and BiB^T^ ranged from ~2.4–3.0 Mb, within the range known for *Orbaceae* (Table 1). The largest contigs for all strains except for lpD03 were circular, representing the main chromosome. The second largest contigs for lpD02^T^ and lpD03 were circular with higher coverage, indicating they are likely plasmids. GenBank Assembly accession numbers are listed in Table 1.

**Table 1.** Genome characteristics of strains lpD01^T^, lpD02^T^, lpD03, lpD04^T^, and BiB^T^ compared to previously described species of the family *Orbaceae* and *Escherichia coli* ATCC 11775^T^

Strain	Genome size (Mb)	CDS	G+C content (%)	Contigs	tRNAs	rRNAs	N50	Busco (%)	CheckM (%)	NCBI assembly
lpD01^T^	2.4	2231	41.3	1	48	14	2 386 378	97	99	GCA_036251705.1
lpD02^T^	2.9	2618	37.2	2	50	13	2 838 661	97	99	GCA_036251875.1
lpD03	3.0	2690	36.2	5	49	13	2 993 133	98	99	GCA_036326945.1
lpD04^T^	2.9	2574	36.0	1	50	13	2 890 389	98	99	GCA_036251935.1
BiB^T^	2.9	2818	35.7	1	55	13	2 925 206	97	99	GCA_036251205.1
*Frischella japonica* Ac13^T^	2.7	2490	34.5	40	48	4	219 086	97	98	GCA_014489845.1
*Frischella perrara* PEB0191^T^	2.7	2375	34.1	1	53	13	2 692 351	98	98	GCA_000807275.1
*Gilliamella apicola* wkB1^T^	3.1	2883	33.6	1	52	12	3 139 412	98	99	GCA_000599985.1
*Gilliamella apicola* wkB7	2.9	2583	34.0	1	52	12	2 901 642	98	99	GCA_001693435.1
*Gilliamella apis* NO3^T^	2.5	2383	34.7	54	43	6	100 334	98	99	GCA_002142155.1
*Gilliamella bombi* LMG 29879^T^	2.6	2609	34.6	163	42	3	55 982	98	99	GCA_900103255.1
*Gilliamella bombicola* LMG 28359^T^	2.3	2225	35.9	34	45	4	113 150	97	98	GCA_900094945.1
*Gilliamella intestini* LMG 28358^T^	2.5	2539	34.6	123	44	2	46 484	97	99	GCA_900094935.1
*Gilliamella mensalis* LMG 29880^T^	2.3	2155	35.5	46	42	3	167 669	97	98	GCA_900103085.1
*Orbus hercynius* CN3^T^	2.4	2180	38.8	16	45	4	395 466	97	99	GCA_003634275.1
*Ca.* Schmidhempelia bombi Bimp	2.2	2067	36.4	131	51	16	38 219	95	97	GCA_000471645.3
*Zophobihabitans entericus* IPMB12^T^	2.7	2384	39.3	2	46	13	2 634 037	98	99	GCA_011745665.1
*Escherichia coli* ATCC 11775^T^	5.0	4984	50.6	2	86	22	4 903 501	100	100	GCA_003697165.2

**Fig. 2.**
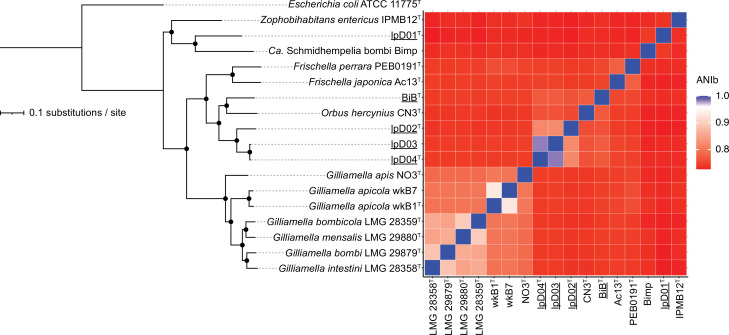
Maximum likelihood phylogeny of *Orbaceae* based on 856 single copy orthologs and corresponding average nucleotide identity (ANIb) values. Accession numbers for assemblies are available in Table 1. Nodes with 100% bootstrap support are indicated with a black circle. The phylogeny scale bar represents 0.1 nucleotide substitutions per site. ANIb values below the suggested species cutoff of 96% are indicated in shades of red, and ANIb values at or above the suggested species cutoff are indicated in white or blue, respectively.

The genomic G+C content ranged from 35.7–41.3%, falling within the range of ~34.0–40.0% characteristic of other members of the family *Orbaceae* (Table 1). lpD01^T^ had six 5S, four 16S, and six 23S rRNA genes. lpD02^T^, lpD03, lpD04^T^, and BiB^T^ had five 5S, four 16S, and six 23S rRNA genes. In all pairwise genomic comparisons between the novel strains and previously-described *Orbaceae*, values were below the species-level cutoff (ANIb/AAI <96.0%, dDDH <70.0%, TI<99.8%; Figs 2, S1, available in the online version of this article). Though lpD01^T^ is most closely related to *Ca.* Schmidhempelia bombi Bimp (Figs 1, 2), pairwise comparisons suggest that this strain represents a distinct, novel genus (ANI=74.3 %, AAI=71.3%). This suggestion is also based on emerging patterns in similarity indices for previously-described *Orbaceae*, as those classified as the same genus share ANI and AAI values ≥75.0% or higher. Pairwise comparisons between lpD03 and lpD04^T^ indicate that these strains belong to the same novel species (dDDH=84.1%, ANIb=98.3%, AAI=97.8%; Figs 2, S1). Strains lpD02^T^, lpD03, lpD04^T^, and BiB^T^ are more closely related to *Orbus hercynius* CN3^T^ (75.8, 76.5, 76.4, and 77.2% ANI, respectively) than to other described *Orbaceae*, suggesting they should be placed as novel species within the genus *Orbus*.

Genes encoding type IVa pili, type Va secretion systems, and type VI secretion systems, subtype i were shared by the presently-described strains and *O. hercynius* CN3^T^ (Fig. S2) [6]. Complete systems not detected included flagella, mannose-sensitive hemagglutinin pili (MSH), type II secretion systems, type III secretion systems, type IV secretion systems subtypes B, C, F, G, I, T, type IVb pili, type VI secretion systems subtypes ii and iii, type IX secretion systems, and type IV protein secretion system subtype i.

A table containing complete KEGG modules identified in anvi’o using anvi-estimate-metabolism for all *Orbaceae* type strains, *G. apicola* wkB7, and lpD03 is available in Table S1. Genes with notable differences between host order groups are shown in Table 2. Pangenomic analysis of *Orbaceae* revealed 8 638 groups of orthologous genes (orthogroups; referred to as ‘gene clusters’ in anvi’o), 983 of which were core clusters (conservatively defined as those present in all *Orbaceae* assemblies included in the analysis), 3 288 of which were present in more than one genome (‘accessory clusters’), and 4 367 of which were unique (Fig. S3). Eleven orthogroups, defined on the basis of homology, were ubiquitous across the six fly-associated *Orbaceae* and absent from all other genomes included in the analysis (‘core dipteran’; Fig. S3, Table S2). These orthogroups putatively encode genes involved in energy production and conversion, translation, ribosomal structure and biogenesis, cell wall/membrane/envelope biogenesis, inorganic ion transfer and metabolism, defence mechanisms, and proteins of unknown or generally-predicted functions. Of these, certain orthogroups encoding stalled ribosome alternative rescue factor (ArfA) and a specific acetyltransferase (Eis) according to COG annotation were only found in all six fly-associated *Orbaceae* genomes. Orthologous genes encoding the catalytic subunit of an aromatic ring-opening dioxygenase (LigB family) were found exclusively in fly-associated *Orbaceae*, though a separate orthogroup annotated with the same function was also identified in the genome of *Z. entericus* IPMB12^T^, isolated from a beetle. According to Pfam annotation, all compared *Orbaceae* genomes contained a single-copy gene cluster putatively encoding a FUSC-like inner membrane protein (YccS) potentially involved in resistance to fusaric acid, but five additional gene clusters in the fusaric acid resistance protein family were exclusively found in fly-associated *Orbaceae*. Two COG-annotated penicillin V acyclase or related amidase, Ntn superfamily (YxeI) protein-coding orthogroups were found exclusively in fly-associated *Orbaceae*. Penicillin and fusaric acid are antimicrobial secondary metabolite produced by fungi in the genera *Penicillium* and *Fusarium,* respectively [72,73]; an expanded resistance strategy to mycotoxins may reflect exposure to these toxins due to the saprophagous diet of many Diptera.

**Table 2.** Select genes annotated with NCBI COGs in anvi’o and sorted and assigned enrichment scores with the programme anvi-compute-functional-enrichment-in-pan and grouped according to host order. Select genes with enrichment scores >9 and unadjusted *p*-values <0.01 are shown Strains: 1, lpD01^T^; 2, lpD02^T^; 3, lpD03; 4, lpD04^T^; 5, BiB^T^; 6, *Orbus hercynius* CN3^T^; 7, *Frischella japonica* Ac13^T^; 8, *Frischella perrara* PEB0191^T^; 9, *Gilliamella apicola* wkB1^T^; 10, *Gilliamella apicola* wkB7; 11, *Gilliamella apis* NO3^T^; 12, *Gilliamella bombi* LMG 29879^T^; 13, *Gilliamella bombicola* LMG 28359^T^; 14, *Gilliamella intestini* LMG 28358^T^; 15, *Gilliamella mensalis* LMG 29880^T^; 16, *Ca.* Schmidhempelia bombi Bimp; 17, *Zophobihabitans entericus* IPMB12^T^. COG categories: C, Energy production and conversion; E, Amino acid transport and metabolism; H, Coenzyme transport and metabolism; J, Translation, ribosomal structure and biogenesis; K, Transcription; M, Cell wall/membrane/envelope biogenesis; N, Cell motility; O, Posttranslational modification, protein turnover, chaperones; P, Inorganic ion transport and metabolism; S, Function unknown; U, Intracellular trafficking, secretion, and vesicular transport; V, Defence mechanisms; W, Extracellular structures. +, Present; –, absent.

COG category	Genes putatively encoding	1	2	3	4	5	6	7	8	9	10	11	12	13	14	15	16	17
C	Citrate lyase synthetase (CitC)	+	+	+	+	+	+	+	+	+	+	+	+	+	+	+	+	−
C	Citrate lyase, alpha subunit (CitF)	+	+	+	+	+	+	+	+	+	+	+	+	+	+	+	+	−
C	Citrate lyase beta subunit (CitE)	+	+	+	+	+	+	+	+	+	+	+	+	+	+	+	+	−
C	Acyl-carrier protein (citrate lyase gamma subunit) (CitD)	+	+	+	+	+	+	+	+	+	+	+	+	+	+	+	+	−
C	Na+/H+-dicarboxylate symporter (GltP)	+	+	+	+	+	+	+	+	+	+	+	+	+	+	+	+	−
C	FMN-dependent NADH-azoreductase (AzoR)	+	+	+	+	+	+	−	−	−	−	−	−	−	−	−	−	+
C, O, P	Nitrate reductase assembly protein (NarJ)	+	+	+	+	+	+	−	−	−	−	−	−	−	−	−	+	+
C, P	Nitrate reductase gamma subunit (NarI)	+	+	+	+	+	+	−	−	−	−	−	−	−	−	−	+	+
C, P	Nitrate reductase alpha subunit (NarG)	+	+	+	+	+	+	−	−	−	−	−	−	−	−	−	+	+
C, P	Nitrate reductase beta subunit (NarY)	+	+	+	+	+	+	−	−	−	−	−	−	−	−	−	+	+
E	Tryptophanase (TnaA)	−	−	−	−	−	−	−	−	−	−	−	−	−	−	−	−	+
E	Urease subunits (UreA-C)	+	−	+	+	−	+	−	−	−	−	−	−	−	−	−	−	−
H	6-pyruvoyl-tetrahydropterin synthase (QueD)	−	−	−	−	−	−	+	+	+	+	+	+	+	+	+	+	−
J	Stalled ribosome alternative rescue factor (ArfA)	+	+	+	+	+	+	−	−	−	−	−	−	−	−	−	−	−
K, N	Negative regulator of flagellin synthesis (anti-sigma28 factor) (FlgM)	−	−	−	−	−	−	+	+	+	+	+	+	+	+	+	−	−
M	Penicillin V acylase or related amidase, Ntn superfamily	+	+	+	+	+	+	−	−	−	−	−	−	−	−	−	−	−
M, O, V	Uncharacterized lipoprotein NlpE involved in copper resistance (CutF), Heat shock protein (HslJ)	−	+	+	+	+	+	−	−	−	−	−	−	−	−	−	−	−
N	Pilin (type one fimbrial protein) (FimA)	+	+	+	+	+	+	−	−	−	−	−	−	−	−	−	−	−
N	Flagellar biosynthesis proteins (FlgA-N)	−	−	−	−	−	−	+	+	+	+	+	+	+	+	+	−	−
N	Flagellar biosynthesis proteins (FlhA and B)	−	−	−	−	−	−	+	+	+	+	+	+	+	+	+	−	−
N	Flagellar motor component (MotA)	−	−	−	−	−	−	+	+	+	+	+	+	+	+	+	−	−
N	Flagellar motor protein (MotB)	−	−	−	−	−	−	+	+	+	+	+	+	+	+	+	−	+
N, U	Flagellar biosynthesis proteins FliA-G	−	−	−	−	−	−	+	+	+	+	+	+	+	+	+	−	−
N, W	Outer membrane usher protein (FimD/PapC)	+	+	+	+	+	+	−	−	−	−	−	−	−	−	−	−	+
O	Urease accessory proteins (UreE,F,H)	+	−	+	+	−	+	−	−	−	−	−	−	−	−	−	−	−
P	Nitrous oxide reductase accessory protein, contains tandem CASH domains (NosD)	−	−	−	−	−	−	−	−	−	−	−	−	−	−	−	−	+
P	Cation transport regulator (ChaB)	−	−	−	−	−	−	+	+	+	+	+	+	+	+	+	+	−
P	Zinc transporter (ZupT)	+	+	+	+	+	+	−	−	−	−	−	−	−	−	−	+	+
P	Alkaline phosphatase (PhoA)	+	−	+	+	+	+	−	−	−	−	−	−	−	−	−	−	−
S	Predicted periplasmic protein with OB-fold, BOF family (YdeI)	+	+	+	+	+	+	−	−	−	−	−	−	−	−	−	−	+
V	Chloramphenicol O-acetyltransferase (CatA)	−	−	−	−	−	−	−	−	−	−	−	−	−	−	−	−	+
w	P pilus assembly protein, chaperone (FimC/PapD)	+	+	+	+	+	+	−	−	−	−	−	−	−	−	−	−	+

### Physiology and biochemical characteristics

**Fig. 3.**
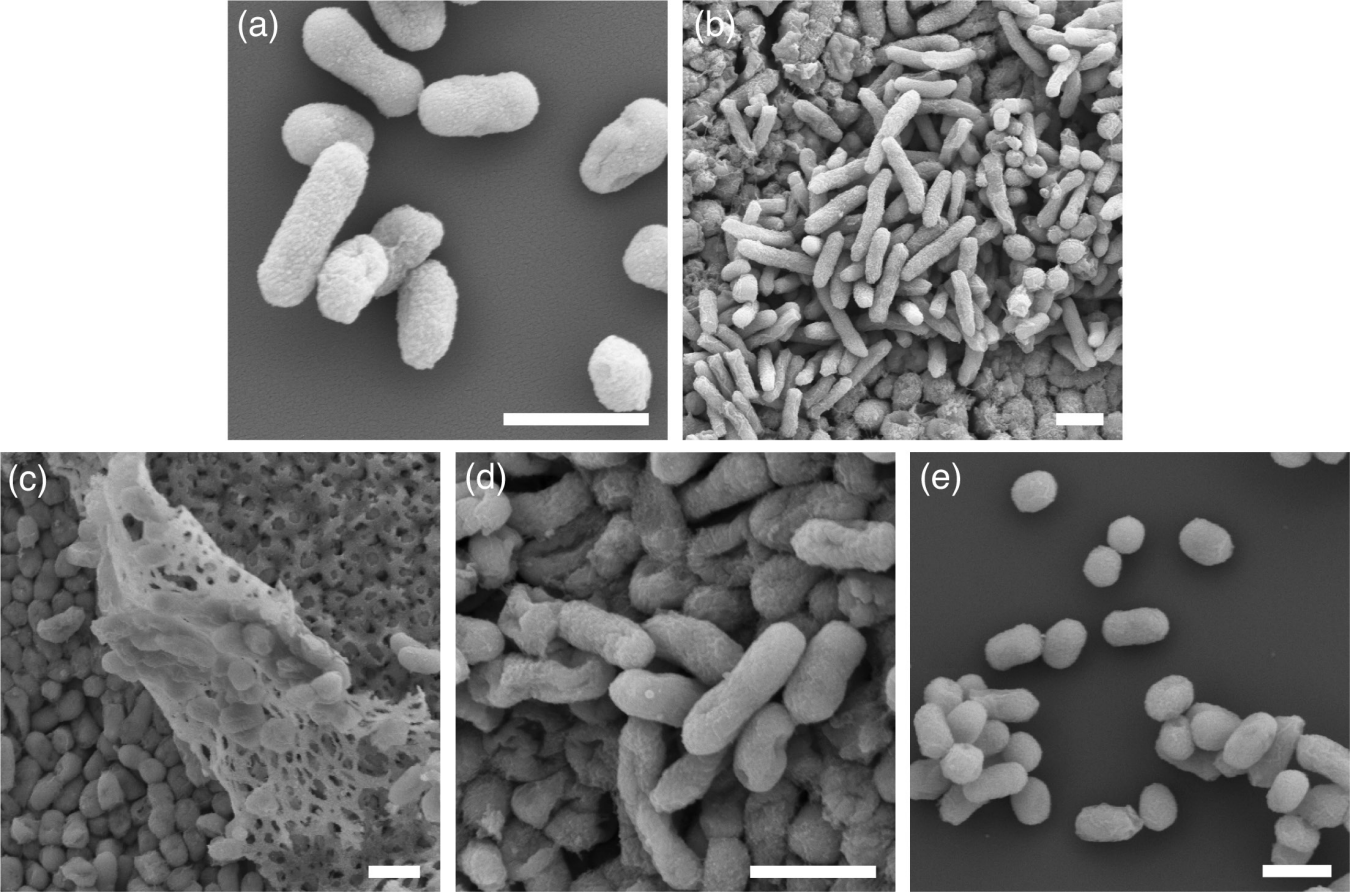
Scanning electron micrographs of (a), lpD01^T^; (b), lpD02^T^; (c), lpD03; (d), lpD04^T^; and (e), BiB^T^ grown for 2 days at 30 °C. Scale bar=1.0 µm.

Cells of lpD01^T^, lpD02^T^, lpD03, lpD04^T^, and BiB^T^ were generally rod-shaped, but appeared more coccoid in certain contexts (Fig. 3). lpD01^T^ commonly measured ~1.6 µm long and ~0.5 wide. Cells of lpD02^T^ commonly measured ~2.0 µm long and ~0.3 µm wide. lpD03 and lpD04^T^ commonly measured ~1.3 µm long and ~0.5 µm wide. Some cells of lpD03 and lpD04^T^ grown in CB displayed long filamentous morphologies, similar to observations of *O. hercynius* CN3^T^ made by Volkmann *et al.* (Fig. S4). BiB^T^ commonly measured ~0.8 µm long and ~0.5 µm wide when grown on HIAb but appeared noticeably longer when grown in CB at a common measurement of ~1.5 µm long.

Optimal growth conditions are summarized in Table 3. In liquid culture, growth was observed for all strains in all tested media except for MB, and very poor growth was observed for all strains in M9 minimal media (OD_600_ <0.001, slightly turbid wells). Summarized results from growth experiments indicated lpD01^T^ grew best in BHI, CB, FAB, Insectagro, and 2X YTD, moderately in LB, and poorly in LIB, TB, and TSB. lpD02^T^ grew best in BHI, CB, and 2X YTD, moderately in FAB and Insectagro, and poorly in LB, LIB, TB, and TSB. lpD03^T^ grew best in BHI, CB, and 2X YTD, moderately in FAB, Insectagro, LIB, TB, and TSB, and poorly in LB. lpD04^T^ grew best in BHI, CB, and 2X YTD, and moderately in FAB, Insectagro, LB, LIB, TB, and TSB. BiB^T^ grew best in BHI, CB, and 2X YTD, moderately in Insectagro, LB, and TSB, and poorly in FAB, LIB, and TB. Optimal growth was observed on HIAb and Columbia agar +5% v/v defibrinated sheep’s blood (CAb) for all of the presently-described strains, and all five strains grew moderately when blood was omitted from HIA and Columbia agar. lpD01^T^ grew moderately on Mueller-Hinton (MH) agar, LB agar, and TSA, and poorly on BHI agar. lpD02^T^ grew poorly on TSA and very poorly on MH, HIA, and LB. lpD03 grew moderately on TSA and BHI and poorly on LB and MH. lpD04 grew poorly on MH, LB, and BHI, and very poorly on TSA. BiB^T^ grew moderately on MH, LB, BHI, and TSA. Strains lpD01^T^, lpD02^T^, and lpD03 were capable of growth between 10–35 °C, lpD04^T^ was capable of growth between 10–30 °C, and BiB^T^ was capable of growth between 10–40 °C. The optimal temperature range was 25–30 °C for lpD01^T^, lpD02^T^, lpD03, and lpD04^T^ and 25–35 °C for BiB^T^. All five strains grew well in atmospheric O_2_ and 5% CO_2_ incubators, but less optimally under anaerobic conditions. Differences in growth with or without agitation were negligible for lpD01^T^ and BiB^T^, lpD02^T^ grew optimally when grown without agitation regardless of the O_2_ concentration, and strains lpD03, and lpD04^T^ grew optimally when shaken at 100 r.p.m. with a slight preference for atmospheric O_2_. Strains lpD01^T^ and lpD03^T^ grew between 0.0–3.0% w/v NaCl, but lpD01^T^ grew optimally at 0.5–2.5% NaCl, and lpD03 grew optimally at 0.5–1.0% NaCl. Strains lpD02^T^ and lpD04^T^ grew between 0.0–2.5% NaCl, but optimally between 0.5–1.0% NaCl. BiB^T^ grew between 0.0–≥3.5% NaCl, but optimally between 0.0–3.0% NaCl. lpD01^T^ grew between pH 5.5–8.5 but optimally at 6.5–7.5 in BHI and between pH ≤6.0–8.5 but optimally between 7.5–8.5 in CB. lpD02^T^ grew between pH 6.0–9.0 but optimally at 7.0–8.5 in BHI and between pH ≤6.0–8.5 but optimally at 7.5–8.5 in CB. lpD03 grew between pH 6.0–8.5 but optimally at 7.0–8.5 in BHI and between pH ≤6.0–8.5 but optimally at 7.5–8.5 in CB. lpD04^T^ grew between pH 5.5–8.0 but optimally at 7.0–7.5 in BHI and between pH ≤6.0–8.5 but optimally at 7.5–8.5 in CB. BiB^T^ grew between pH 5.0–9.5 but optimally at 6.0–6.5 in BHI and between pH ≤6.0–≥9.0 but optimally at 6.5–7.5 in CB.

**Table 3.** Optimal growth conditions for strains lpD01^T^, lpD02^T^, lpD03, lpD04^T^, and BiB^T^. NaCl range is based on summarized results from experiments using BHI and 2X YTD broths Strains: 1, lpD01^T^; 2, lpD02^T^; 3, lpD03; 4, lpD04^T^; 5, BiB^T^. np=no preference was observed between tested conditions.

Optimal condition	1	2	3	4	5
Temperature (°C)	25–30	25–30	25–30	25–30	25–35
Shaking	np	No	Yes	Yes	np
pH (BHI)	6.5–7.5	7.0–8.5	7.0–8.5	7.0–7.5	6.0–6.5
pH (CB)	7.5–8.5	7.5–8.5	7.5–8.5	7.5–8.5	6.5–7.5
NaCl (%)	0.5–2.5	0.5–1.0	0.5–1.0	0.5–1.0	0.0–3.0
Agar	HIAb/CAb	HIAb/CAb	HIAb/CAb	HIAb/CAb	HIAb/CAb
Broth	BHI/CB/FAB/2X YTD	BHI/CB/2X YTD	BHI/CB/2X YTD	BHI/CB/2X YTD	BHI/CB/2X YTD

Glycerol stocks or lyophilized cultures are both acceptable forms of cryopreservation for these strains. The strains remained viable following lyophilization in ATCC Medium 9520 (10% w/v BSA and 20% w/v sucrose) and storage for 72 h at room temperature. The revived lyophilized cultures of lpD01^T^, lpD02^T^, lpD03, and BiB^T^ contained ~10^7^ CFU ml^−1^, whereas lpD04^T^ retained a viability of ~10^6^ CFU ml^−1^.

Results from motility assays suggest that the presently-described strains are motile, but we cannot confidently determine their precise method of motility. Initially, after 5 days of incubating the motility agar assay at 30 °C, results indicated a lack of motility due to a lack of diffused growth outside the stab line. However, we observed considerable diffusion approximately 1 week later, indicating that these strains are potentially motile. A repeat of this experiment yielded the same results. In subsequent experiments using semisolid agar a wide zone of diffuse growth was not observed for lpD01^T^, lpD02^T^, lpD03, lpD04^T^, and BiB^T^ compared to *E. coli* ATCC 25922; however, a multi-ring-like, star-shaped colony morphology with a transparent perimeter formed after approximately 2–4 days. This transparent ring around the site of inoculation measured approximately 2.0 cm in diameter for BiB^T^ and 1.5 cm for lpD01^T^, noticeably larger than those observed for lpD02^T^, lpD03, and lpD04^T^, which were ~0.6 cm in diameter. For twitching motility assays wide, star-shaped surface colonies and interstitial colonies, both with a highly asymmetrical morphology, were observed for all of the presently-described strains. These results were similar to those observed for *O. hercynius* CN3^T^, concluded to be motile by an undetermined method by Volkmann *et al.* [6].

lpD01^T^, lpD02^T^, lpD03, and BiB^T^ did not exhibit noticeable biofilm formation under tested conditions; however, a ‘mesh-like’ substrate was observed in multiple samples, including lpD03, during scanning electron microscopic analysis (Fig. 3).

A summary of biochemical characteristics is available in Table 4. Strains lpD03 and lpD04^T^ were oxidase-positive, and strains lpD01^T^, lpD02^T^, and BiB^T^ were delayed oxidase-positive. All strains were positive for catalase activity. All of the presently-described strains except for lpD02 exhibited d-glucose and d-mannose fermentation in assays performed using the Microgen GN ID-A kit. lpD01^T^, lpD03, and lpD04^T^ exhibited urease activity, which correlated perfectly with the presence of urease genes in urease-positive organisms and absence in urease-negative ones. All other Microgen GN ID-A test results were negative. Interestingly, the presently-described organisms encode citrate lyases and nitrate reductases, indicating the potential for relevant biochemical activities, though they were not observed using this testing method. Results from TSI slant cultures indicated all strains were positive for glucose and lactose and/or sucrose fermentation, and tests for lpD03 and lpD04^T^ indicated the possible production of gas based on observed bubbles and cracks in the agar. The conflicting glucose fermentation results for lpD02^T^ are possibly due to an incompatibility of this strain with the Microgen GN ID-A microwell contents. lpD01^T^, lpD02^T^, lpD03, lpD04^T^, and BiB^T^ did not exhibit DNase activity.

**Table 4.** Differential biochemical characteristics of the five newly isolated strains and close relatives in the family *Orbaceae* Strains: 1, lpD01^T^; 2, lpD02^T^; 3, lpD03; 4, lpD04^T^; 5, BiB^T^; 6, *Frischella japonica* Ac13^T^; 7, *Frischella perrara* PEB0191^T^; 8, *Gilliamella apicola* wkB1^T^; 9, *Gilliamella apis* NO3^T^; 10, *Gilliamella bombi* LMG 29879^T^; 11, *Gilliamella bombicola* LMG 28359^T^; 12, *Gilliamella intestini* LMG 28358^T^; 13, *Gilliamella mensalis* LMG 29880^T^; 14, *Orbus hercynius* CN3^T^; 15, *Orbus sasakiae* C7^T^; 16, *Zophobihabitans entericus* IPMB12^T^. +, Positive; d+, delayed positive; –, negative; w, weakly positive; v, variable; nd, no data.

Characteristic	1	2	3	4	5	6	7	8	9	10	11	12	13	14	15	16
Acetoin production	−	−	−	−	−	nd	nd	nd	+	nd	nd	nd	nd	nd	nd	nd
Catalase	+	+	+	+	+	−	+	−	−	+	+	−	−	+	+	−
Citrate utilization	−	−	−	−	−	nd	nd	nd	nd	nd	nd	nd	nd	−	nd	+
Glucose fermentation	+	+	+	+	+	+	v	v	+	−	−	−	−	+	+	+
Mannitol fermentation	+	−	+	+	+	+	−	nd	−	nd	nd	nd	nd	nd	+	+
Xylose fermentation	−	−	−	−	−	nd	+	nd	nd	nd	nd	nd	nd	nd	+	nd
DNAse	−	−	−	−	−	nd	nd	nd	nd	−	−	−	−	nd	nd	−
Indole production	−	−	−	−	−	−	−	−	−	nd	nd	nd	nd	−	−	+
Lactose and/or sucrose fermentation	+	+	+	+	+	+	−	nd	+	−	−	nd	−	nd	+	+
Lysine decarboxylase	−	−	−	−	+	nd	nd	nd	nd	nd	nd	nd	nd	nd	nd	nd
Nitrate reductase	−	−	−	−	−	−	−	−	nd	−	−	−	−	+	+	+
β-Galactosidase	−	−	−	−	+	−	−	+	−	−	−	+	−	−	+	nd
Ornithine decarboxylase	−	−	−	−	−	nd	nd	nd	nd	nd	nd	nd	nd	nd	−	+
Oxidase	d+	d+	+	+	d+	−	−	−	−	−	−	−	−	+	−	+
Tryptophan deaminase	−	−	−	−	−	nd	nd	nd	nd	−	−	−	−	nd	nd	nd
Urease	+	−	+	+	−	−	−	−	−	−	−	−	−	+	−	−

Strain lpD04^T^ exhibited the following MIC: >800 µg ml^−1^ carbenicillin, 6.25 µg ml^−1^ chloramphenicol, 25 µg ml^−1^ kanamycin, 50 µg ml^−1^ spectinomycin, and 12.5 µg ml^−1^ tetracycline. MIC for lpD01^T^, lpD02^T^, lpD03, and BiB^T^ were previously reported in Elston *et al.* [26]. The summarized MIC results are available in Table S3.

Strain lpD04^T^ was successfully genetically engineered to express Pathfinder plasmids pSL1 and pSL1-GFP [26] via conjugation.

### Chemotaxonomy

The major fatty acids (>10%) for lpD01^T^ were summed feature 8, C_16 : 0_, summed feature 3, and C_14 : 0_. Major fatty acids for lpD02^T^, lpD03, and BiB^T^ were summed feature 8 (C_18 : 1_* ω*6*c* and/or C_18 : 1_* ω*7*c*), C_16 : 0_, and summed feature 3 (C_16 : 1_* ω6*c and/or C_16 : 1_* ω7*c). Major fatty acids for lpD04^T^ were summed feature 8 and C_16 : 0_. A comparison with 11 previously-described *Orbaceae* shows that all currently-described strains share the same major fatty acids, summed feature 8 and C_16 : 0_, and differ notably in compositions of C_12 : 0_, C_14 : 0_, summed feature 2 (C_16 : 0_ isomer I and/or C_14 : 0_ 3OH), summed feature 3, C_18 : 0_, and/or C_18 : 1_* ω*9*c* (Table S4).

The major respiratory quinone for lpD01^T^ was ubiquinone 8 (Q-8; 99.3%), and trace amounts of ubiquinone 9 were detected (0.7%).

## Discussion

Isolation and characterization of the presently-described strains expands the repertoire of culturable bacteria naturally associated with insects. Results from phylogenetic and genomic analyses suggest that strain lpD01^T^ belongs to a novel genus, *Utexia*, and strains lpD02^T^, lpD03, lpD04^T^, and BiB^T^ belongs to novel species in the genus *Orbus.* We propose the names *Utexia brackfieldae* gen. nov., sp. nov. lpD01^T^, *Orbus sturtevantii* sp. nov. lpD02^T^, *Orbus wheelerorum* sp. nov. lpD04^T^, and *Orbus mooreae* sp. nov. BiB^T^. These bacteria are amenable to genetic engineering and capable of colonizing lab-reared *D. melanogaster* [26] and are thus promising subjects for investigation of host-microbe interactions in this model organism. In conclusion, this description enriches our understanding of insect microbiota and presents a valuable resource for fine-scale exploration of host-microbe dynamics within the controlled environment of lab-reared *D. melanogaster*.

## Description of *Utexia* gen. nov.

*Utexia brackfieldae* (U.tex’i.a. N.L. fem. n. *Utexia*, arbitrary name formed from the contraction of The University of Texas at Austin, where descriptive studies of this taxon were performed).

Cells are mesophilic, Gram-negative rods. Optimal growth conditions are reached under atmospheric O_2_ conditions, but growth is also observed in anaerobic conditions and 5% CO_2_. The predominant respiratory quinone is Q-8. The major fatty acids are *cis*-vaccenic acid (summed feature 8; C_18 : 1_* ω*6*c* and/or C_18 : 1_* ω*7*c*) and palmitic acid (C_16 : 0_). Based on 16S rRNA gene and single-copy ortholog phylogenetic analysis, the genus is most closely related to *Ca.* Schmidhempelia and *Zophobihabitans*. The type species of the genus is *Utexia brackfieldae*.

## Description of *Utexia brackfieldae* sp. nov.

*Utexia brackfieldae* (brack.fiel’dae N.L. gen. n. *brackfieldae*, arbitrary epithet formed from a contraction of Brackenridge Field Laboratory, the research station where the fly from which this taxon was first isolated was caught).

In addition to those given in the genus description, *Utexia brackfieldae* exhibits the following characteristics. Strains grow optimally on blood agar, but are capable of growth on TSA, HIA, LBA, 2X YTD agar, Columbia, and DNase agar. They form smooth, round, off-white colonies with a transparent perimeter approximately 4 mm in diameter or smaller after 3 days of incubation at 30 °C. Strains grow between 10–35 °C, but optimally at 25–30 °C. In BHI, the growth range is pH 5.5–8.5 (optimal range 6.5–7.5), and in CB, the growth range is pH ≤6.0–8.5 (optimal range 7.5–8.5). In 2X YTD broth, the growth range is 0.0–3.0% w/v NaCl (optimal range 0.5–2.5%). Cells are approximately 1.6 by 0.5 µm. Cultures were delayed-positive for oxidase, and positive for glucose/dextrose fermentation, mannitol fermentation, lactose and/or sucrose fermentation, urease, and catalase. They were negative for acetoin production, citrate utilization, nitrate reductase, lysine decarboxylase, ornithine decarboxylase, hydrogen sulphide, xylose fermentation, β-galactosidase, indole production, tryptophan deaminase, and DNAse activity. The main constituent fatty acids are *cis*-vaccenic acid (36.4%, C_18 : 1_* ω*6*c* and/or C_18 : 1_* ω*7*c*) and palmitic acid (35.1%, C_16 : 0_).

The type strain is lpD01^T^ (=NCIMB 15517^T^ =ATCC TSD-399^T^) isolated from a wild-caught *Neogriphoneura sordida* in Austin, Texas, USA. The GenBank accession numbers for the 16S rRNA gene and whole genome sequence are OR490959 and CP133959, respectively. The genome assembly accession number and name are GCA_036251705.1 and ASM3625170v1, respectively.

## Description of *Orbus sturtevantii* sp. nov.

*Orbus sturtevantii* (stur.te.van’ti.i. N. L. gen. n. *sturtevantii*, named after Alfred Henry Sturtevant, American fly geneticist, professor, and describer of *Drosophila cardini*, the fly species from which this bacterium was isolated).

In addition to those given in the genus description, *O. sturtevantii* exhibits the following characteristics. Strains grow optimally on blood agar, but are capable of growth on TSA, HIA, LBA, 2X YTD agar, Columbia, and DNase agar. They form smooth, round, off-white colonies with a transparent perimeter approximately 3 mm in diameter or smaller after 5 days of incubation at 30 °C. Strains grow between 10–35 °C, but optimally at 25–30 °C. In BHI, the growth range is pH 6.0–9.0 (optimal range 7.0–8.5), and in CB, the growth range is pH ≤6.0–8.5 (optimal range 7.5–8.5). In 2X YTD broth, the growth range is 0.0–2.5% w/v NaCl (optimal range 0.5–1.0%). Cells are approximately 2.0 µm by 0.3 µm. Cultures were delayed-positive for oxidase, and positive for glucose/dextrose fermentation, mannitol fermentation, lactose and/or sucrose fermentation, urease, and catalase. They were negative for nitrate reductase, lysine decarboxylase, ornithine decarboxylase, hydrogen sulphide, xylose fermentation, ONPG hydrolysis, indole production, acetoin, citrate utilization, tryptophan deaminase, and DNAse activity. The main constituent fatty acids are palmitic acid (C_16 : 0_) and *cis*-vaccenic acid (C_18 : 1_* ω*6*c* and/or C_18 : 1_* ω*7*c*).

The type strain is lpD02^T^ (=NCIMB 15518^T^ =ATCC TSD-400^T^) isolated from wild-caught *Drosophila cardini* in Austin, Texas, USA. The GenBank accession numbers for the 16S rRNA gene and whole genome sequence are OR490960 and CP133960-CP133961, respectively. The genome assembly accession number and name are GCA_036251875.1 and ASM3625187v1, respectively.

## Description of *Orbus wheelerorum* sp. nov.

*Orbus wheelerorum* (whee.le.ro’rum. N.L. gen. pl. n. *wheelerorum*, ‘of Wheelers,’ named after William Morton Wheeler and Marshall R. Wheeler, American biologists, naturalists, and professors who performed research on insects at the University of Texas at Austin, USA).

In addition to those given in the genus description, *O. wheelerorum* exhibits the following characteristics. Strains grow optimally on blood agar, but are also capable of growth on TSA, HIA, LBA, 2X YTD agar, Columbia, and DNase agar. They form smooth, round, off-white colonies with a transparent perimeter approximately 2.5 mm in diameter or smaller after 3 days of incubation at 30 °C. Strains grow between 10–30 °C for lpD04^T^ (10–35 °C for lpD03), but optimally at 25–30 °C. For lpD04^T^ the growth range is pH 5.5–8.0 (optimal range 7.0–7.5) in BHI, and pH ≤6.0–8.5 (optimal range 7.5–8.5) in CB. In 2X YTD broth, the growth range is 0.0–2.0% w/v NaCl for type strain lpD04^T^ (optimal range 0.5–1.0%). Cells are approximately 1.3 µm by 0.5 µm (Fig. 3). Strains are positive for oxidase, glucose/dextrose fermentation, mannitol fermentation, lactose and/or sucrose fermentation, urease, and catalase, are negative for nitrate reductase, lysine decarboxylase, ornithine decarboxylase, hydrogen sulphide, xylose fermentation, ONPG hydrolysis, indole production, acetoin, citrate utilization, tryptophan deaminase, and DNAse activity. The main constituent fatty acids are *cis*-vaccenic acid (C_18 : 1_* ω*6*c* and/or C_18 : 1_* ω*7*c*) and palmitic acid (C_16 : 0_).

The type strain is lpD04^T^ (=NCIMB 15520^T^ =ATCC TSD-401^T^) isolated from a wild-caught male *Drosophila melanogaster* in Austin, Texas, USA. The GenBank accession numbers for the 16S rRNA gene and whole genome sequence of lpD04^T^ are OR490962 and CP133967, respectively. The genome assembly accession number and name are GCA_036251935.1 and ASM3625193v1, respectively. Another strain of this species, lpD03 (=NCIMB 15519=ATCC BAA-3339), was isolated from a wild-caught *Drosophila meridiana* in Austin, Texas, USA. The GenBank accession numbers for the 16S rRNA gene and whole genome sequence of lpD03 are OR490961 and CP133962-CP133966, respectively. The genome assembly accession number and name are GCA_036326945.1 and ASM3632694v1, respectively.

## Description of *Orbus mooreae* sp. nov.

*Orbus mooreae* (moo’re.ae. N.L. gen. n. *mooreae*, named after Ruth Ella Moore, microbiologist, professor, and the first Black American woman to receive a PhD in the Natural Sciences [74]).

In addition to those given in the genus description, *O. mooreae* exhibits the following characteristics. Strains grow optimally on blood agar, but are capable of growth on TSA, HIA, LBA, 2X YTD agar, Columbia, and DNase agar. They form smooth, round, off-white colonies with a transparent perimeter approximately 3.5 mm in diameter or smaller after 3 days of incubation at 30 °C. Strains grow between 10–40 °C, but optimally at 25–35 °C. In BHI, the growth range is pH 5.0–9.5 (optimal range 6.0–6.5), and in CB, the growth range is pH ≤6.0-≥9.0 (optimal range 6.5–7.5). In 2X YTD broth, the growth range is 0–≥3.5 % w/v NaCl, with an optimal range of 0.0–3.0%. Cells are approximately 0.8 µm by 0.5 µm on HIAb, and 1.5 µm long in CB. Cultures were delayed-positive for oxidase, and positive for lysine decarboxylase, glucose/dextrose fermentation, mannitol fermentation, lactose and/or sucrose fermentation, β-galactosidase, and catalase. They were negative for nitrate reductase, ornithine decarboxylase, hydrogen sulphide, xylose fermentation, indole production, urease, acetoin, citrate utilization, tryptophan deaminase, and DNAse activity. The main constituent fatty acids are *cis*-vaccenic acid (45.8%, C_18 : 1_* ω*6*c* and/or C_18 : 1_* ω*7*c*) and palmitic acid (36.6%, C_16 : 0_).

The type strain is BiB^T^ (=NCIMB 15516^T^ =ATCC TSD-402^T^) isolated from a wild-caught *Drosophila* sp. in Austin, Texas, USA. The GenBank accession numbers for the 16S rRNA gene and whole genome sequence are OR490958 and CP133958, respectively. The genome assembly accession number and name are GCA_036251205.1 and ASM3625120v1, respectively.

## supplementary material

10.1099/ijsem.0.006516 Uncited Supplementary Material 1.

## Abbreviations

AAI: average amino acid identity
ANI: average nucleotide identity
ANIb: average nucleotide identity based on BLASTN+ alignments
BHI: brain heart infusion
BLAST: Basic Local Alignment Search Tool
BOLD: Barcode of Life Data System
CAb: Columbia agar + 5% v/v defibrinated sheep's blood
CB: Columbia broth
CFU: colony-forming unit
COG: Cluster of Orthologous Group of proteins
COI: cytochrome oxidase I
COII: cytochrome oxidase II
FAB: fastidious anaerobe broth
GGDC: Leibniz Institute DSMZ Genome to Genome Distance Calculator
GSAF: The University of Texas at Austin Genomic Sequencing and Analysis Facility
HIA: heart infusion agar
HIAb: heart infusion agar + 5% v/v defibrinated sheep's blood
HMDS: hexamethyldisilazane
KEGG: Kyoto Encyclopedia of Genes and Genomes
KO: Kyoto Encyclopedia of Genes and Genomes ortholog
LB: lysogeny broth or Luria-Bertani
LIB: liver infusion broth
MB: marine broth
MH: Mueller-Hinton
MSH: mannose-sensitive hemagglutinin pili
NCBI: National Center for Biotechnology Information
ONT: Oxford Nanopore Technologies
SEM: scanning electron microscopy
TB: tryptose broth
TI: tetranucleotide identity
TSB: trypticase soy broth
TSI: triple sugar iron
TTC: 2,3,5-triphenyltetrazolium chloride
YT: yeast extract tryptone
YTD: yeast extract tryptone + 0.3% w/v dextrose
