# Supplementary material for: Characterization of gut symbionts from wild-caught Drosophila and other Diptera: description of Utexia brackfieldae gen. nov., sp. nov., Orbus sturtevantii sp. nov., Orbus wheelerorum sp. nov, and Orbus mooreae sp. nov
Source: Int J Syst Evol Microbiol. 2024 Sep 27;74(9):006516. doi: 10.1099/ijsem.0.006516 (PMC11434166; doi:10.1099/ijsem.0.006516)
Supplement: Uncited Supplementary Material 1. [file ijsem-74-06516-s001.pdf]

Supplementary Materials for:

Characterization of gut symbionts from wild-caught *Drosophila* and other Diptera: Description of *Utexia brackfieldae* gen. nov., sp. nov., *Orbus sturtevantii* sp. nov., *Orbus wheelerorum* sp. nov, and *Orbus mooreae* sp. nov.

Laila E. Phillips[1*], Kathleen L. Sotelo[1], Nancy A. Moran[1]
[1]Department of Integrative Biology, The University of Texas at Austin, Austin, TX 78712, USA
[*]Correspondence: lphillips@utexas.edu

This PDF file includes:

- **Supplementary Figures S1 to S4**
- **Supplementary Tables S1 to S4**
- **Supplementary Material References**

Supplementary Figures

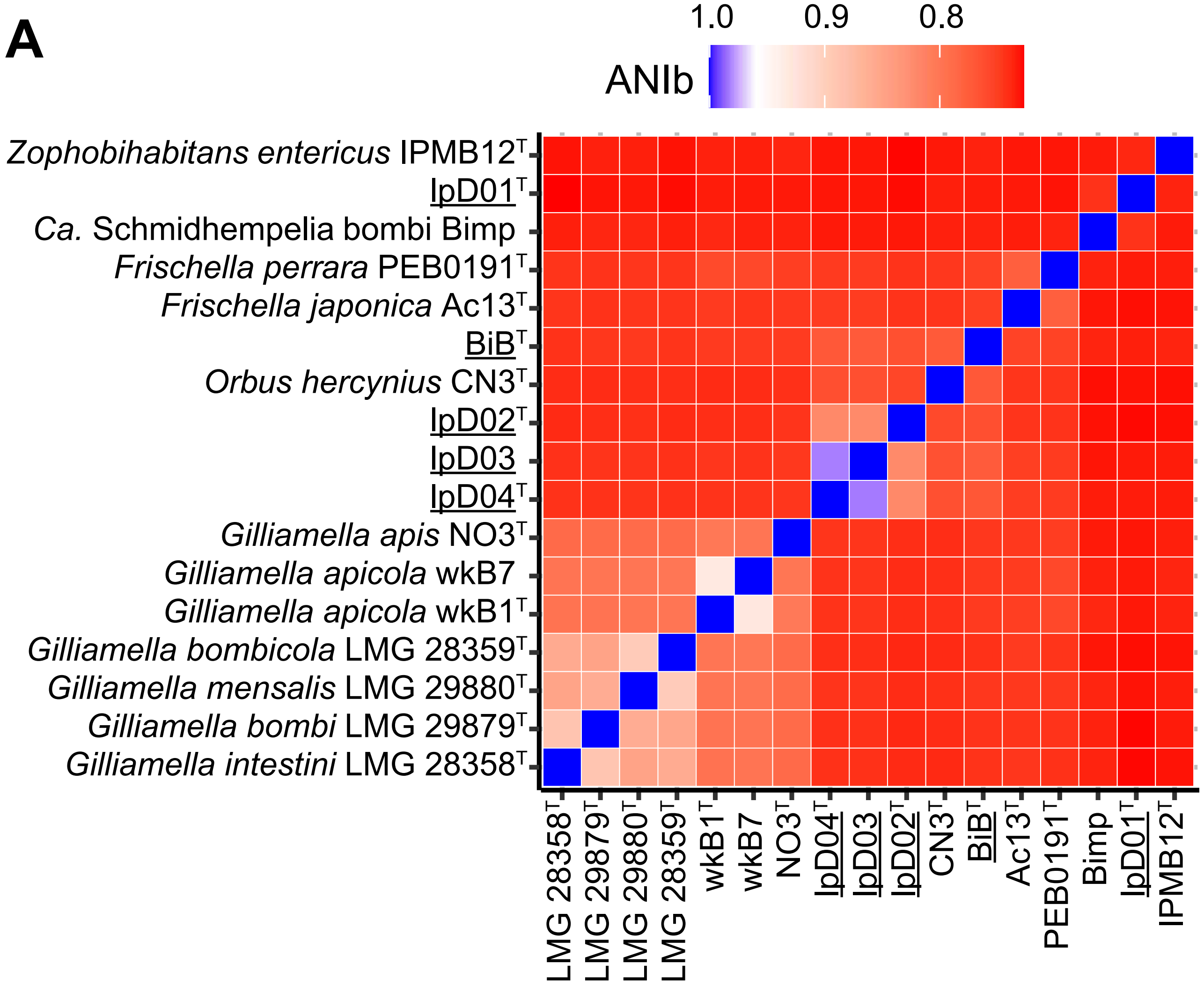

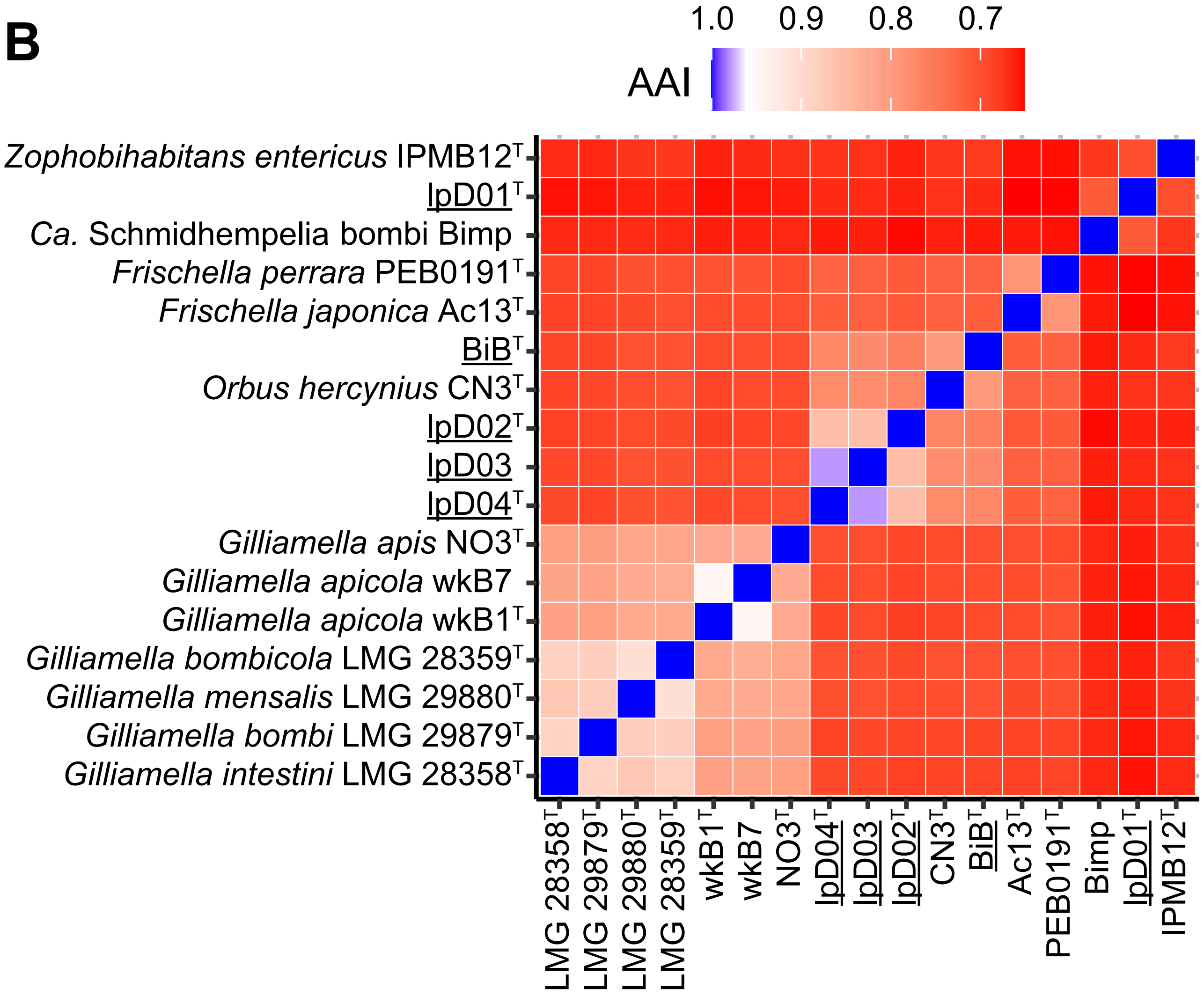
B
1.0
0.9
0.8
0.7
AAI
Zophobihabitans entericus IPMB12T
IpD01T
Ca. Schmidhempelia bombi Bimp
Frischella perrara PEB0191T
Frischella japonica Ac13T
BiBT
Orbus hercynius CN3T
IpD02T
IpD03
IpD04T
Gilliamella apis NO3T
Gilliamella apicola wkB7
Gilliamella apicola wkB1T
Gilliamella bombicola LMG 28359T
Gilliamella mensalis LMG 29880T
Gilliamella bombi LMG 29879T
Gilliamella intestini LMG 28358T
LMG 28358T
LMG 29879T
LMG 29880T
LMG 28359T
wkB1T
wkB7
NO3T
IpD04T
IpD03
IpD02T
CN3T
BiBT
Ac13T
PEB0191T
Bimp
IpD01T
IPMB12T

C

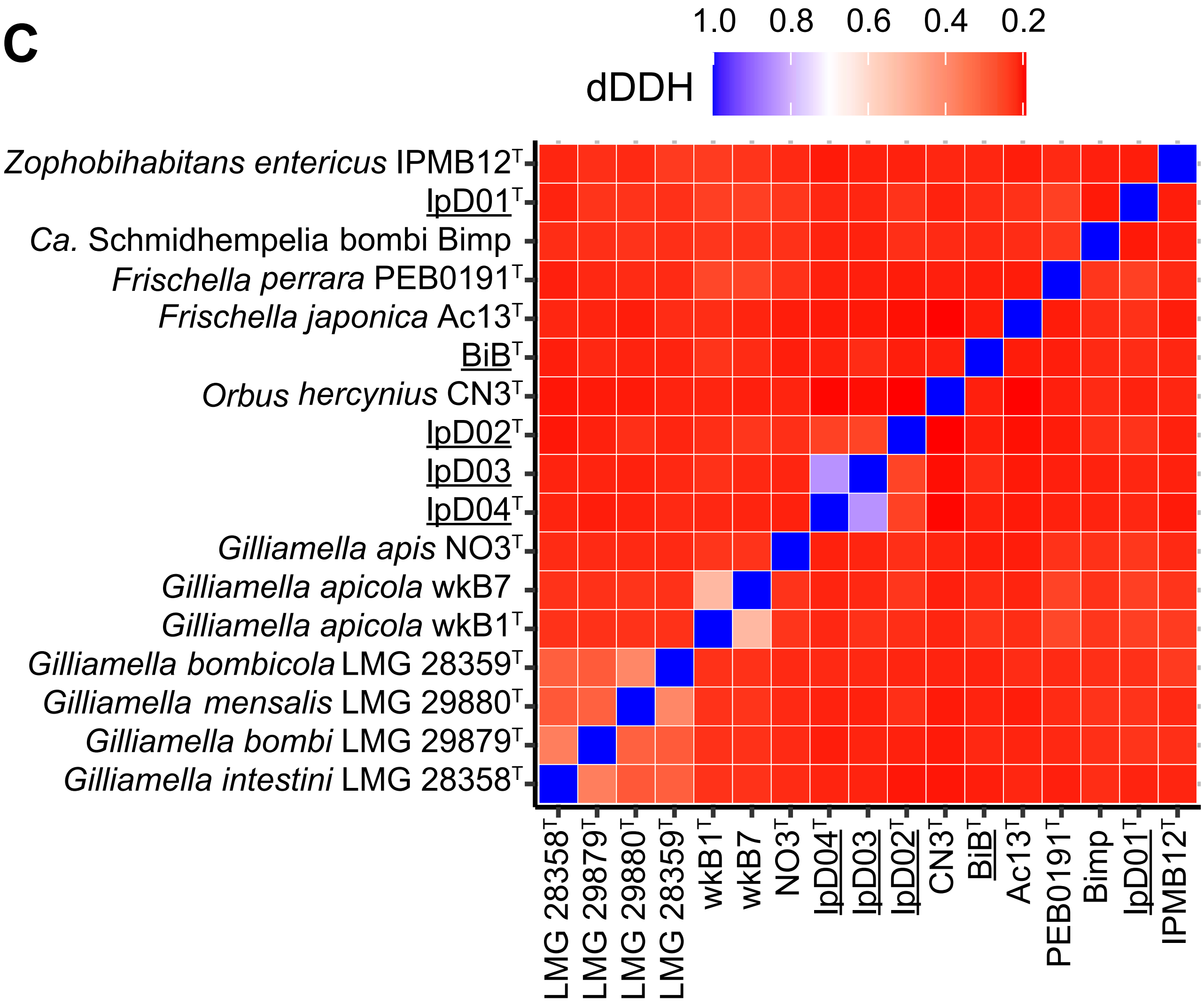

1.0 0.8 0.6 0.4 0.2
dDDH
Zophobihabitans entericus IPMB12T
IpD01T
Ca. Schmidhempelia bombi Bimp
Frischella perrara PEB0191T
Frischella japonica Ac13T
BiBT
Orbus hercynius CN3T
IpD02T
IpD03
IpD04T
Gilliamella apis NO3T
Gilliamella apicola wkB7
Gilliamella apicola wkB1T
Gilliamella bombicola LMG 28359T
Gilliamella mensalis LMG 29880T
Gilliamella bombi LMG 29879T
Gilliamella intestini LMG 28358T
LMG 28358T
LMG 29879T
LMG 29880T
LMG 28359T
wkB1T
wkB7
NO3T
IpD04T
IpD03
IpD02T
CN3T
BiBT
Ac13T
PEB0191T
Bimp
IpD01T
IPMB12T

D

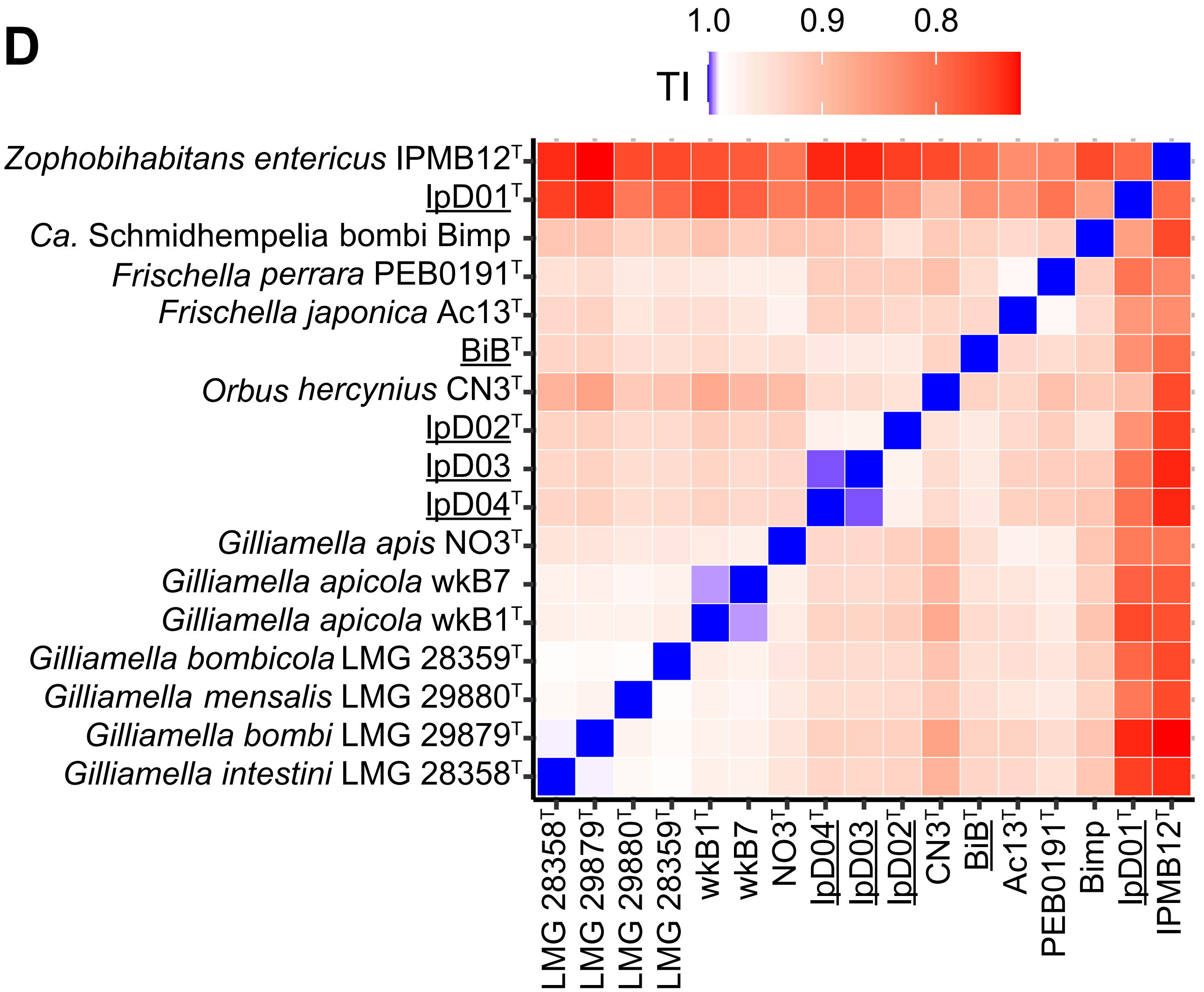

Supplementary Figure S1. Genome-wide similarity comparisons of available *Orbaceae* genomes. Accession numbers for genome assemblies are indicated in Table 1 of main text. (A) ANIb (B) AAI (C) dDDH (D) TI. Pairwise comparison values below suggested cutoff values for species classification for each method (ANIb/AAI < 96%, dDDH < 70%, TI < 99.8%) are indicated in red, and values at or above suggested species cutoffs are indicated in white or blue, respectively.

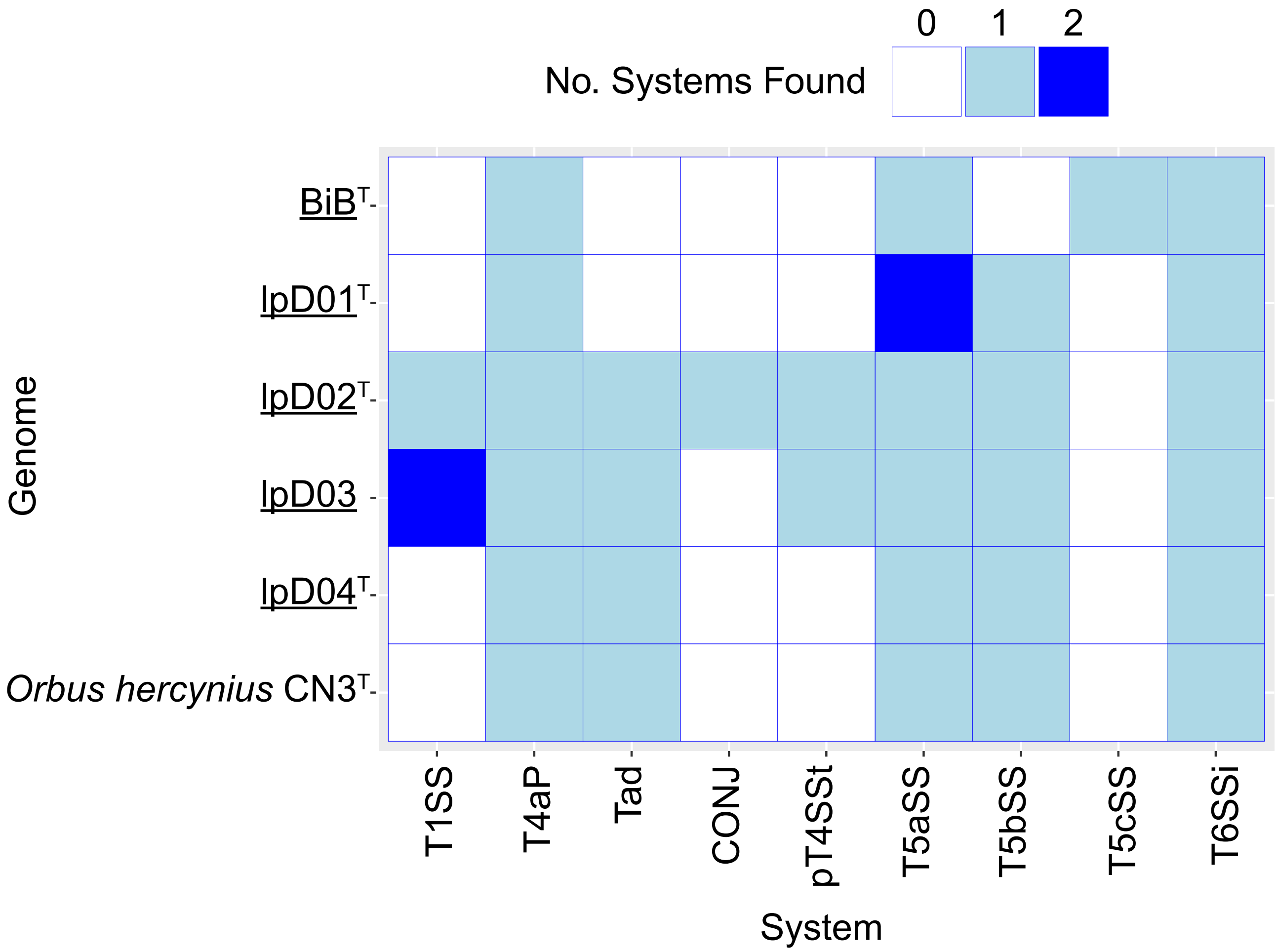

Supplementary Figure S2. Complete secretion systems identified in RAST-annotated genome assemblies for strains BiB^T, lpD01^T, lpD02^T, lpD03, lpD04^T, and *Orbus hercynius* CN3^T (1) using MacSyFinder (v. 2.0) via the TXSScan model (v. 1.1.0) and stringent default settings (2–6). T1SS, type I secretion system; T4aP, type IV pili subtype a; Tad, tight adherence pili; CONJ, conjugation system; pT4SSt, type IV protein secretion system subtype t; T5aSS, type V secretion system subtype a (autotransporter); T5bSS, type V secretion system subtype b (two-partner); T5cSS, type V secretion system subtype c (trimeric); T6SSi, type VI secretion system subtype i.

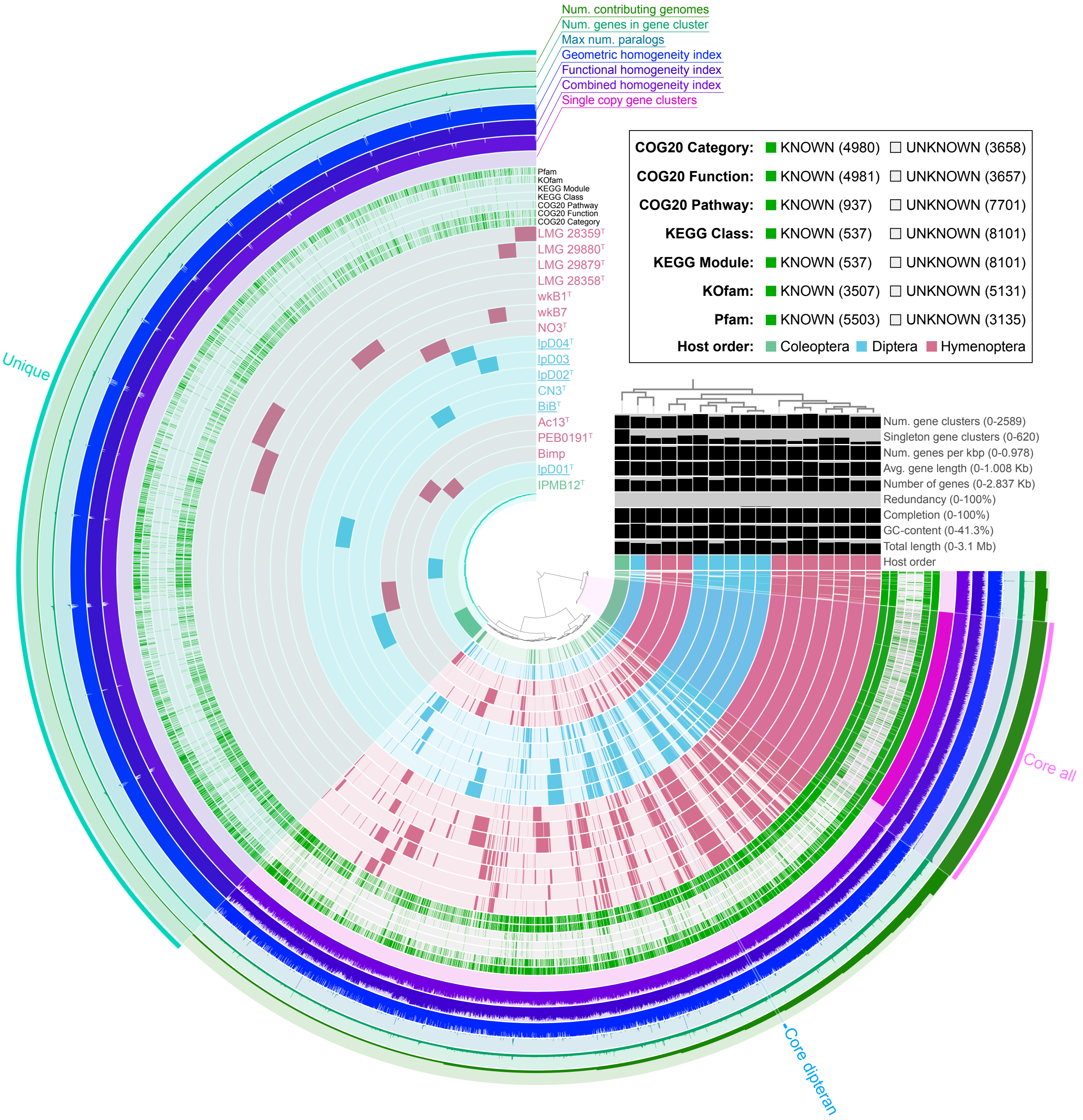

Supplementary Figure S3. A pangenome phylogram for 17 strains of *Orbaceae* prepared using the anvi'o programs anvi-pan-genome and anvi-display-pan (7). Each circular bar represents a genome colored according to the order of the host from which the strain was isolated, with darker regions indicating gene presence. Orthogroups (referred to as "gene clusters" in anvi'o) are ordered according to presence-absence. The total number of orthogroups unique to each strain ("singletons") and those common to all strains ("core") are indicated; unlabeled orthogroups are present in more than one, but not all, assemblies. The similarities in gene content among strains associated with the same host order may suggest evolutionary specialization and host specificity.

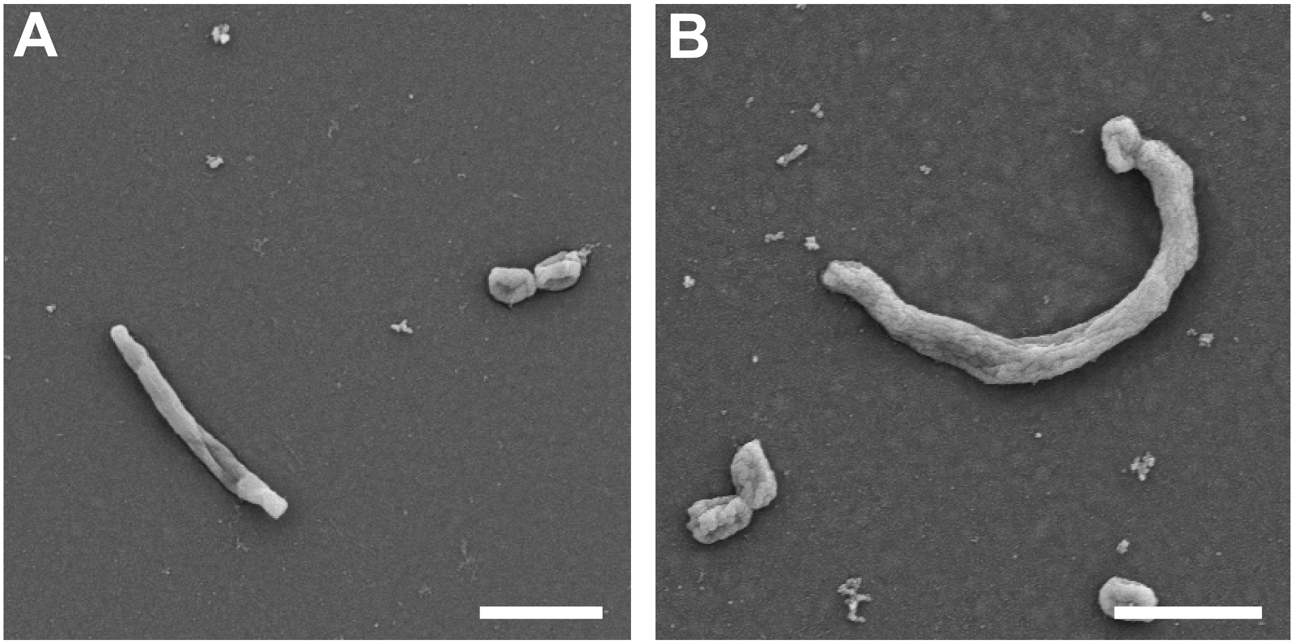

Supplementary Figure S4. Scanning electron micrographs of strains (A) lpD03 and (B) lpD04^{T} demonstrating a filamentous morphology only observed under liquid culture conditions during our experiments. SEM was performed on cultures grown in Columbia broth as described in the main text. Scale bar = 2.0 µm.

Supplementary Tables

Supplementary Table S1. Kyoto Encyclopedia of Genes and Genomes (KEGG) modules indicated as complete (“1”, ≥75% KOs) or incomplete (“0”, <75% KOs) via the anvi’o program anvi-estimate-metabolism (7). Strains: 1, lpD01^{T}; 2, lpD02^{T}; 3, lpD03; 4, lpD04^{T}; 5, BiB^{T}; 6, *Orbus hercynius* CN3^{T}; 7, *Frischella japonica* Ac13^{T}; 8, *Frischella perrara* PEB0191^{T}; 9, *Gilliamella apicola* wkB1^{T}; 10, *Gilliamella apicola* wkB7; 11, *Gilliamella apis* NO3^{T}; 12, *Gilliamella bombi* LMG 29879^{T}; 13, *Gilliamella bombicola* LMG 28359^{T}; 14, *Gilliamella intestini* LMG 28358^{T}; 15, *Gilliamella mensalis* LMG 29880^{T}; 16, *Ca.* Schmidhempelia bombi Bimp; 17, *Zophobihabitans entericus* IPMB12^{T}.

Module	Module Name	1	2	3	4	5	6	7	8	9	10	11	12	13	14	15	16	17
M00001	Glycolysis (Embden-Meyerhof pathway), glucose => pyruvate	1	1	1	1	1	1	1	1	1	1	1	1	1	1	1	1	1
M00002	Glycolysis, core module involving three-carbon compounds	1	1	1	1	1	1	1	1	1	1	1	1	1	1	1	1	1
M00003	Gluconeogenesis, oxaloacetate => fructose-6P	1	1	1	1	1	1	1	1	1	1	1	1	1	1	1	1	1
M00004	Pentose phosphate pathway (Pentose phosphate cycle)	1	1	1	1	1	1	1	1	1	1	1	1	1	1	1	1	1
M00005	PRPP biosynthesis, ribose 5P => PRPP	1	1	1	1	1	1	1	1	1	1	1	1	1	1	1	1	1
M00006	Pentose phosphate pathway, oxidative phase, glucose 6P => ribulose 5P	1	1	1	1	1	1	1	1	1	1	1	1	1	1	1	1	1
M00007	Pentose phosphate pathway, non-oxidative phase, fructose 6P => ribose 5P	1	1	1	1	1	1	1	1	1	1	1	1	1	1	1	1	1
M00008	Entner-Doudoroff pathway, glucose-6P => glyceraldehyde-3P + pyruvate	1	1	1	1	1	1	0	0	1	1	1	1	1	1	1	0	1
M00015	Proline biosynthesis, glutamate => proline	1	1	1	1	1	1	1	1	1	1	1	1	1	1	1	0	1
M00016	Lysine biosynthesis, succinyl-DAP pathway, aspartate => lysine	1	1	1	1	1	1	1	1	1	1	1	1	1	1	1	1	1
M00017	Methionine biosynthesis, apartate => homoserine => methionine	1	1	1	1	1	1	1	1	1	1	1	1	1	1	1	1	1
M00018	Threonine biosynthesis, aspartate => homoserine => threonine	1	1	1	1	1	1	1	1	1	1	1	1	1	1	1	1	1
M00019	Valine/isoleucine biosynthesis, pyruvate => valine / 2-oxobutanoate => isoleucine	1	1	1	1	1	1	1	1	1	1	1	1	1	1	1	1	1
M00020	Serine biosynthesis, glycerate-3P => serine	1	1	1	1	1	1	1	1	1	1	1	1	1	1	1	1	1
M00021	Cysteine biosynthesis, serine => cysteine	1	1	1	1	1	1	1	1	1	1	1	1	1	1	1	0	1

Module	Module Name	1	2	3	4	5	6	7	8	9	10	11	12	13	14	15	16	17
M00022	Shikimate pathway, phosphoenolpyruvate + erythrose-4P => chorismate	1	1	1	1	1	1	1	1	1	1	1	1	1	1	1	1	1
M00023	Tryptophan biosynthesis, chorismate => tryptophan	1	1	1	1	1	1	1	1	1	1	1	1	1	1	1	1	1
M00024	Phenylalanine biosynthesis, chorismate => phenylpyruvate => phenylalanine	1	1	1	1	1	1	1	1	1	1	1	1	1	1	1	1	0
M00025	Tyrosine biosynthesis, chorismate => HPP => tyrosine	1	1	1	1	1	1	1	1	1	1	1	1	1	1	1	1	0
M00026	Histidine biosynthesis, PRPP => histidine	1	1	1	1	1	1	1	1	1	1	1	1	1	1	1	1	1
M00027	GABA (gamma-Aminobutyrate) shunt	0	0	1	1	0	0	0	0	0	0	0	0	0	0	0	0	0
M00028	Ornithine biosynthesis, glutamate => ornithine	1	1	1	1	1	1	1	1	1	1	1	1	1	1	1	1	1
M00048	Inosine monophosphate biosynthesis, PRPP + glutamine => IMP	1	1	1	1	1	1	1	1	1	1	1	1	1	1	1	1	1
M00049	Adenine ribonucleotide biosynthesis, IMP => ADP,ATP	1	1	1	1	1	1	1	1	1	1	1	1	1	1	1	1	1
M00050	Guanine ribonucleotide biosynthesis IMP => GDP,GTP	1	1	1	1	1	1	1	1	1	1	1	1	1	1	1	1	1
M00051	Uridine monophosphate biosynthesis, glutamine (+ PRPP) => UMP	1	1	1	1	1	1	0	0	1	1	0	0	0	0	0	0	1
M00052	Pyrimidine ribonucleotide biosynthesis, UMP => UDP/UTP,CDP/CTP	1	1	1	1	1	1	0	0	1	1	1	1	1	1	0	1	1
M00053	Pyrimidine deoxyribonuleotide biosynthesis, CDP/CTP => dCDP/dCTP,dTDP/dTTP	1	1	1	1	1	1	1	1	1	1	1	1	1	1	1	1	1
M00060	KDO2-lipid A biosynthesis, Raetz pathway, LpxL-LpxM type	1	1	1	1	1	1	1	1	1	1	1	1	1	1	1	1	1
M00061	D-Glucuronate degradation, D-glucuronate => pyruvate + D-glyceraldehyde 3P	0	1	1	0	1	0	0	0	1	1	1	0	1	1	1	0	1
M00063	CMP-KDO biosynthesis	1	1	1	1	1	1	1	1	1	1	1	1	1	1	1	1	1
M00064	ADP-L-glycero-D-manno-heptose biosynthesis	1	1	1	1	1	1	1	1	1	1	1	1	1	1	1	1	1
M00082	Fatty acid biosynthesis, initiation	1	1	1	1	1	1	1	1	1	1	1	1	1	1	1	1	1
M00083	Fatty acid biosynthesis, elongation	1	1	1	1	1	1	1	1	1	1	1	1	1	1	1	1	1
M00093	Phosphatidylethanolamine (PE) biosynthesis, PA => PS => PE	1	1	1	1	1	1	1	1	1	1	1	1	1	1	1	1	1
M00096	C5 isoprenoid biosynthesis, non-mevalonate pathway	1	1	1	1	1	1	1	1	1	1	1	1	1	1	1	1	1

Module	Module Name	1	2	3	4	5	6	7	8	9	10	11	12	13	14	15	16	17
M00098	Acylglycerol degradation	0	0	0	0	0	0	1	0	0	0	0	0	0	0	0	0	0
M00118	Glutathione biosynthesis, glutamate => glutathione	1	1	1	1	1	1	1	1	1	1	1	1	1	1	1	1	1
M00120	Coenzyme A biosynthesis, pantothenate => CoA	1	1	1	1	1	1	1	1	1	1	1	1	1	1	1	1	1
M00121	Heme biosynthesis, plants and bacteria, glutamate => heme	1	1	1	1	1	1	1	1	1	1	1	1	1	1	1	1	1
M00125	Riboflavin biosynthesis, plants and bacteria, GTP => riboflavin/FMN/FAD	1	1	1	1	1	1	1	1	1	1	1	1	1	1	1	1	1
M00126	Tetrahydrofolate biosynthesis, GTP => THF	1	1	1	1	1	1	1	1	1	1	1	1	1	1	1	1	1
M00140	C1-unit interconversion, prokaryotes	1	1	1	1	1	1	1	1	1	1	1	1	1	1	1	0	1
M00150	Fumarate reductase, prokaryotes	0	0	0	0	1	0	0	0	0	0	0	0	0	0	0	0	1
M00153	Cytochrome bd ubiquinol oxidase	1	0	0	0	0	0	0	0	1	1	1	0	0	0	1	0	1
M00157	F-type ATPase, prokaryotes and chloroplasts	1	1	1	1	1	1	1	1	1	1	1	1	1	1	1	1	1
M00167	Reductive pentose phosphate cycle, glyceraldehyde-3P => ribulose-5P	1	0	0	0	1	1	0	0	0	0	1	0	0	0	0	0	1
M00176	Assimilatory sulfate reduction, sulfate => H2S	0	1	1	1	1	1	0	1	1	1	0	0	0	0	0	0	0
M00307	Pyruvate oxidation, pyruvate => acetyl-CoA	1	1	1	1	1	1	1	1	1	1	1	1	1	1	1	1	1
M00308	Semi-phosphorylative Entner-Doudoroff pathway, gluconate => glycerate-3P	1	1	1	1	1	1	0	0	1	1	1	1	1	1	1	0	1
M00344	Formaldehyde assimilation, xylulose monophosphate pathway	0	0	0	0	1	1	0	0	0	0	0	0	0	0	0	0	1
M00345	Formaldehyde assimilation, ribulose monophosphate pathway	0	1	0	0	1	0	0	0	1	0	1	0	1	0	1	0	1
M00432	Leucine biosynthesis, 2-oxoisovalerate => 2-oxoisocaproate	1	1	1	1	1	1	1	1	1	1	1	1	1	1	1	1	1
M00525	Lysine biosynthesis, acetyl-DAP pathway, aspartate => lysine	0	0	0	0	1	0	0	0	0	0	0	0	0	0	0	0	0
M00526	Lysine biosynthesis, DAP dehydrogenase pathway, aspartate => lysine	1	1	1	1	1	1	1	1	1	1	1	1	1	1	1	1	1
M00527	Lysine biosynthesis, DAP aminotransferase pathway, aspartate => lysine	1	1	1	1	1	1	1	1	1	1	1	1	1	1	1	1	1
M00530	Dissimilatory nitrate reduction, nitrate => ammonia	1	1	1	1	1	1	0	0	0	0	0	0	0	0	0	1	1
M00549	Nucleotide sugar biosynthesis, glucose => UDP-glucose	0	0	0	0	1	0	0	1	0	0	0	0	0	0	0	0	0
M00550	Ascorbate degradation, ascorbate => D-xylulose-5P	1	1	1	1	1	1	1	1	1	1	1	0	0	1	1	1	0

Module	Module Name	1	2	3	4	5	6	7	8	9	10	11	12	13	14	15	16	17
M00552	D-galactonate degradation, De Ley-Doudoroff pathway, D-galactonate => glycerate-3P	0	1	0	0	1	0	0	0	0	0	0	0	0	0	0	0	1
M00554	Nucleotide sugar biosynthesis, galactose => UDP-galactose	0	0	0	0	1	0	0	0	1	1	0	1	0	1	1	0	0
M00570	Isoleucine biosynthesis, threonine => 2-oxobutanoate => isoleucine	1	1	1	1	1	1	1	1	1	1	1	1	1	1	1	1	1
M00579	Phosphate acetyltransferase-acetate kinase pathway, acetyl-CoA => acetate	1	1	1	1	1	1	1	1	1	1	1	1	1	1	1	1	1
M00580	Pentose phosphate pathway, archaea, fructose 6P => ribose 5P	0	0	0	0	0	0	0	0	0	0	0	0	0	0	0	0	1
M00616	Sulfate-sulfur assimilation	0	1	1	1	1	1	0	1	1	1	0	0	0	0	0	0	0
M00631	D-Galacturonate degradation (bacteria), D-galacturonate => pyruvate + D-glyceraldehyde 3P	1	1	1	1	1	1	0	0	1	1	1	0	1	0	1	0	1
M00632	Galactose degradation, Leloir pathway, galactose => alpha-D-glucose-1P	0	0	0	0	1	0	0	0	1	1	0	1	0	1	1	0	0
M00702	Multidrug resistance, efflux pump NorB	0	0	0	0	1	0	0	0	1	1	1	0	0	0	0	0	1
M00793	dTDP-L-rhamnose biosynthesis	1	0	0	0	1	0	1	0	1	1	1	0	0	1	0	0	0
M00844	Arginine biosynthesis, ornithine => arginine	1	1	1	1	1	1	1	1	1	1	1	1	1	1	1	1	1
M00846	Siroheme biosynthesis, glutamyl-tRNA => siroheme	1	1	1	1	1	1	1	1	1	1	1	1	1	1	1	1	1
M00866	KDO2-lipid A biosynthesis, Raetz pathway, non-LpxL-LpxM type	1	1	1	1	1	1	1	1	1	1	1	1	1	1	1	1	1
M00880	Molybdenum cofactor biosynthesis, GTP => molybdenum cofactor	1	1	1	1	1	1	1	1	1	1	1	1	1	1	1	1	1
M00881	Lipoic acid biosynthesis, plants and bacteria, octanoyl-ACP => dihydrolipoyl-E2/H	1	1	1	1	1	1	1	1	1	1	1	1	1	1	1	1	1
M00899	Thiamine salvage pathway, HMP/HET => TMP	0	0	0	0	1	1	1	1	1	1	1	1	1	1	1	0	1
M00909	UDP-N-acetyl-D-glucosamine biosynthesis, prokaryotes, glucose => UDP-GlcNAc	1	1	1	1	1	1	1	1	1	1	1	1	1	1	1	0	1
M00916	Pyridoxal-P biosynthesis, R5P + glyceraldehyde-3P + glutamine => pyridoxal-P	1	0	0	0	1	1	0	0	1	1	1	1	1	1	1	1	1
M00926	Heme biosynthesis, bacteria, glutamyl-tRNA => coproporphyrin III => heme	1	1	1	1	1	1	1	1	1	1	1	1	1	1	1	1	1

Supplementary Table S2. Eleven orthogroups ("gene clusters") that are ubiquitous across the six fly-associated *Orbaceae* and absent from all other genomes included in the analysis identified using the anvi'o program anvi-pan-genome (7) (See also: Fig. S3).

Gene Cluster ID	COG20 ID	COG20 Category	COG20 Function	KOfam ID	KOfam Function	Pfam ID	Pfam Function
1474	COG3111	S	Predicted periplasmic protein YdeI with OB-fold, BOF family (YdeI)			PF04076.17	Bacterial OB fold (BOF) protein
2010	COG2223	P	Nitrate/nitrite transporter NarK (NarK)	K02532	MFS transporter, OHS family, lactose permease	PF01306.23	LacY proton/sugar symporter
2051	COG3036	J	Stalled ribosome alternative rescue factor ArfA (ArfA)	K09890	alternative ribosome-rescue factor	PF03889.17	Alternative ribosome-rescue factor A
2069	COG1902	C	2,4-dienoyl-CoA reductase or related NADH-dependent reductase, Old Yellow Enzyme (OYE) family			PF00724.24	NADH:flavin oxidoreductase / NADH oxidase family
2076	COG1289	S	Uncharacterized membrane protein YccC (YccC)			PF13515.10	Fusaric acid resistance protein-like
2107	COG1182	C	FMN-dependent NADH-azoreductase (AzoR)	K01118	FMN-dependent NADH-azoreductase [EC:1.7.1.17]	PF02525.21	Flavodoxin-like fold
2115	COG4552	R	Predicted acetyltransferase (Eis)			PF13527.11	Acetyltransferase (GNAT) domain
2129	COG3049	M|R	Penicillin V acylase or related amidase, Ntn superfamily			PF02275.22	Linear amide C-N hydrolases, choloylglycine hydrolase family
2156	COG1566	V	Multidrug resistance efflux pump EmrA (EmrA)	K03543	membrane fusion protein, multidrug efflux system	PF13437.10	HlyD family secretion protein
2177						PF10938.12	YfdX protein
2211	COG3384	Q	Aromatic ring-opening dioxygenase, catalytic subunit, LigB family (LigB)	K15777	4,5-DOPA dioxygenase extradiol [EC:1.13.11.-]	PF02900.22	Catalytic LigB subunit of aromatic ring-opening dioxygenase

Supplementary Table S3. Minimum inhibitory concentrations of carbenicillin (Carb), chloramphenicol (Cam), kanamycin (Kan), spectinomycin (Spec), and tetracycline (Tet) in liquid media for strains lpD01^T, lpD02^T, lpD03, lpD04^T, and BiB^T. Units are in µg/ml. * = Data previously reported in Elston et al. (8).

Strain	Carb	Cam	Kan	Spec	Tet
lpD01^T*	6.25	400	12.5	400	200
lpD02^T*	200	6.25	50	100	6.25
lpD03*	800	6.25	25	100	6.25
lpD04^T	>800	6.25	25	50	12.5
BiB^T*	25	6.25	400	100	6.25

Supplementary Table S4. Fatty acid (%) composition of the newly-described strains and other described species of *Orbaceae*. Data for the following strains are from the following references: *Frischella japonica* Ac13T (9); *Frischella perrara* PEB0191T (10); *Gilliamella apicola* wkB1T (11); *Gilliamella apis* NO3T (12); *Gilliamella bombi* LMG 29879T (13); *Gilliamella bombicola* LMG 28359T (13); *Gilliamella intestini* LMG 28358T (13); *Gilliamella mensalis* LMG 29880T (13); *Orbus hercynius* CN3T (1); *Zophobihabitans entericus* IPMB12T (14). Major fatty acids (>10%) are indicated in bold. Fatty acids comprising lower than 1% for all compared strains were omitted. Fatty acids for wkB1T are marked "other" where specific values were not mentioned in the original reference (11). Fatty acids for NO3T are indicated in descending order of asterisks according to how data were presented in the original reference (12).

**Genome**	$C_{12:0}$	$C_{14:0}$	$C_{16:0}$	**Summed Feature 2 ($C_{16:1}$ isomer I & $C_{14:0}$ 3OH)**	**Summed Feature 3 ($C_{16:1}$ *ω6c* & $C_{16:1}$ *ω7c*)**	$C_{18:0}$	**Summed Feature 8 ($C_{18:1}$ *ω6c* & $C_{18:1}$ *ω7c*)**	$C_{18:1}$ **ω9c**	**other**
lpD01T	0.57	**12.20**	**35.11**	1.82	**12.95**	0.50	**36.43**	-	-
lpD02T	0.08	4.52	**43.74**	1.18	**10.18**	0.80	**38.87**	-	-
lpD03	-	5.76	**34.54**	0.15	**17.84**	0.89	**39.79**	-	-
lpD04T	-	3.45	**36.29**	1.26	5.22	0.64	**52.50**	-	-
BiBT	-	3.67	**36.60**	1.16	**11.10**	0.94	**45.74**	-	-
Ac13T	-	7.50	**42.00**	**10.60**	4.70	2.20	**33.20**	-	-
PEB0191T	-	5.15	**35.05**	9.36	1.96	3.29	**44.41**	0.78	-
wkB1T	other	7.52	**31.69**	other	9.41	1.31	**41.32**	-	8.75
NO3T	-	*	***	**	-	-	****	-	-
LMG 29879T	1.59	9.65	**35.19**	**12.95**	3.56	1.33	**34.75**	0.97	-
LMG 28359T	1.60	8.01	**35.62**	**11.88**	2.33	1.81	**37.03**	1.22	-
LMG 28358T	**23.17**	**13.60**	**27.60**	-	-	-	**18.09**	**17.54**	-
LMG 29880T	7.82	**12.29**	**29.63**	**27.08**	3.43	-	**19.74**	-	-
CN3T	0.16	6.88	**33.73**	9.37	**10.70**	0.35	**38.45**	-	-
C7T	0.60	**11.20**	**28.00**	**19.90**	9.30	<0.50%	**29.40**	-	-
IPMB12T	7.00	**19.10**	**19.30**	5.60	7.00	1.50	**36.00**	-	4.50

Supplementary Material References

1. Volkmann M, Skiebe E, Kerrinnes T, Faber F, Lepka D, Pfeifer Y, Holland G, Bannert N, Wilharm G. 2010. *Orbus hercynius* gen. nov., sp. nov., isolated from faeces of wild boar, is most closely related to members of the orders "*Enterobacteriales*" and *Pasteurellales*. Int J Syst Evol Microbiol 60:2601–2605.
2. Abby SS, Rocha EPC. 2012. The non-flagellar type III secretion system evolved from the bacterial flagellum and diversified into host-cell adapted systems. PLoS Genet 8:e1002983.
3. Abby SS, Néron B, Ménager H, Touchon M, Rocha EPC. 2014. MacSyFinder: A program to mine genomes for molecular systems with an application to CRISPR-Cas systems. PLoS ONE 9:e110726.
4. Abby SS, Cury J, Guglielmini J, Néron B, Touchon M, Rocha EPC. 2016. Identification of protein secretion systems in bacterial genomes. Sci Rep 6:23080.
5. Denise R, Abby SS, Rocha EPC. 2019. Diversification of the type IV filament superfamily into machines for adhesion, protein secretion, DNA uptake, and motility. PLoS Biol 17:e3000390.
6. 2022. TXSScan: Annotation of bacterial protein secretion systems. MacSy Models.
7. Eren AM, Kiefl E, Shaiber A, Veseli I, Miller SE, Schechter MS, Fink I, Pan JN, Yousef M, Fogarty EC, Trigodet F, Watson AR, Esen ÖC, Moore RM, Clayssen Q, Lee MD, Kivenson V, Graham ED, Merrill BD, Karkman A, Blankenberg D, Eppley JM, Sjödin A, Scott JJ, Vázquez-Campos X, McKay LJ, McDaniel EA, Stevens SLR, Anderson RE, Fuessel J, Fernandez-Guerra A, Maignien L, Delmont TO, Willis AD. 2020. Community-led, integrated, reproducible multi-omics with anvi'o. Nat Microbiol 6:3–6.
8. Elston KM, Phillips LE, Leonard SP, Young E, Holley JC, Ahsanullah T, McReynolds B, Moran NA, Barrick JE. 2023. The Pathfinder plasmid toolkit for genetically engineering newly isolated bacteria enables the study of *Drosophila*-colonizing *Orbaceae*. ISME Commun 3.
9. Wolter LA, Suenami S, Miyazaki R. 2019. *Frischella japonica* sp. nov., an anaerobic member of the *Orbales* in the *Gammaproteobacteria*, isolated from the gut of the eastern honey bee, *Apis cerana japonica* Fabricius. Int J Syst Evol Microbiol 71.
10. Engel P, Kwong WK, Moran NA. 2013. *Frischella perrara* gen. nov., sp. nov., a gammaproteobacterium isolated from the gut of the honeybee, *Apis mellifera*. Int J Syst Evol Microbiol 63:3646–3651.
11. Kwong WK, Moran NA. 2013. Cultivation and characterization of the gut symbionts of honey bees and bumble bees: Description of *Snodgrassella alvi* gen. nov., sp. nov., a member of the family *Neisseriaceae* of the *Betaproteobacteri*a, and *Gilliamella apicola* gen. nov., sp. nov., a member of *Orbaceae* fam. nov., *Orbales* ord. nov., a sister taxon to the order '*Enterobacteriales*' of the *Gammaproteobacteria*. Int J Syst Evol Microbiol 63:2008–2018.
12. Ludvigsen J, Porcellato D, Amdam GV, Rudi K. 2018. Addressing the diversity of the honeybee gut symbiont *Gilliamella*: description of *Gilliamella apis* sp. nov., isolated from the gut of honeybees (*Apis mellifera*). Int J Syst Evol Microbiol 68:1762–1770.
13. Praet J, Cnockaert M, Meeus I, Smagghe G, Vandamme P. 2017. *Gilliamella intestini* sp. nov., *Gilliamella bombicola* sp. nov., *Gilliamella bombi* sp. nov. and *Gilliamella mensalis* sp. nov.: Four novel *Gilliamella* species isolated from the bumblebee gut. Syst Appl Microbiol 40:199–204.
14. Kuo C-H, Huang P-Y, Sheu S-Y, Sheu D-S, Jheng L-C, Chen W-M. 2021. *Zophobihabitans entericus* gen. nov., sp. nov., a new member of the family *Orbaceae* isolated from the gut of a superworm *Zophobas morio*. Int J Syst Evol Microbiol 71.
